# Breast cancer and neoplasms of the thyroid gland: a bidirectional two-sample Mendelian randomization study

**DOI:** 10.3389/fonc.2024.1422009

**Published:** 2024-10-14

**Authors:** Yiqi Sun, Bohan Wan, Xin Liu, Jianguo Dong, Shengjie Yin, Yiqi Wu

**Affiliations:** ^1^ Department of Pathology, Harbin Medical University, Harbin, Heilongjiang, China; ^2^ Department of Medical Oncology, Municipal Hospital of Chifeng, Chifeng, Inner Mongolia, China

**Keywords:** breast cancer, thyroid gland neoplasms, cancer epidemiology, Mendelian randomization, cancer genetics

## Abstract

**Background:**

With the rising incidence of breast cancer (BC) and neoplasms of the thyroid gland, a potential link between the two has drawn increasing attention. However, the causal relationship remains unclear due to various confounding factors. This study aims to investigate the causality between BC and thyroid tumors using Mendelian Randomization (MR) analysis.

**Methods:**

We conducted a bidirectional two-sample MR analysis, utilizing breast cancer-associated single nucleotide polymorphisms (SNPs) from the Breast Cancer Association Consortium (BCAC) and thyroid tumor-related SNPs from the FinnGen (https://www.finngen.fi/) database. First, we performed univariable MR (UVMR) to assess the causal relationship between BC and both malignant and benign thyroid tumors, followed by reverse causality analysis. To account for potential confounders, we applied multivariable MR (MVMR). The inverse-variance weighted (IVW) method was primarily used, with secondary analyses performed using the weighted median and MR-Egger regression approaches.

**Results:**

UVMR analysis revealed a significant positive causal relationship between BC and malignant thyroid tumors (odds ratio [OR] and 95% confidence interval [CI]: 1.291, 1.143–1.458, *P* = 3.90×10^-5^). No causal relationship was found between BC and benign thyroid tumors. The MVMR analysis, adjusting for confounding factors such as smoking, drinking, and body mass index (BMI), confirmed the robustness of the results.

**Conclusion:**

This study provides genetic evidence supporting a causal relationship between BC and malignant thyroid tumors. These findings highlight the importance of thyroid cancer screening in BC patients. However, further MR studies or randomized controlled trials (RCTs) are necessary to assess small effects accurately.

## 1 Introduction

BC is the most common malignancy among women and one of the leading cancers globally (1). Its incidence has steadily increased over the past 40 years, with a 0.5% annual rise from 2010 to 2019 (2). BC is classified into several subtypes, including Luminal A, Luminal B, HER2-positive, and triple-negative breast cancer (TNBC) via immunohistochemical method (3). Currently, despite advances in treatment, 20-30% of BC patients develop metastasis, often leading to poor outcomes (4).

Treatment for early-stage BC may involve breast-conserving surgery (BCS) or mastectomy, often followed by breast reconstruction. Radiotherapy is commonly used after BCS, while drug therapies include chemotherapy for high recurrence risk, endocrine therapy for hormone receptor-positive cases, and HER2-targeted therapy for HER2-positive patients (5). Immune checkpoint inhibitors (ICIs) show limited efficacy as monotherapy but improve outcomes when combined with chemotherapy, particularly in metastatic TNBC. Also, biomarkers like PD-L1 expression aid in predicting treatment response (6). However, these treatments may also bring about some issues. Radiotherapy can lead to thyroid disorders, including hypothyroidism, thyroiditis, and thyroid cancer (7). Nowadays, advances in radiotherapy and dose reduction strategies can mitigate these risks, and regular thyroid function monitoring is advised. Compared to radiotherapy, chemotherapy may reduce thyroid nodules incidence by affecting TSH levels (8). However, ICIs can cause thyroid dysfunction, including nodules and neoplasms of the thyroid gland (9).

Neoplasms of the thyroid gland, classified by the WHO as thyroid adenomas, low-risk tumors, and thyroid carcinomas, are increasing in incidence (10). Thyroid carcinoma accounts for 1% of all newly diagnosed cancers annually, with risk factors like obesity contributing to its rising incidence (11, 12). While rare, BC can metastasize to the thyroid, usually indicating advanced disease and poor prognosis. For instance, Ramírez Stieben et al. reported a BC patient with thyroid metastasis confirmed via fine-needle aspiration biopsy (FNAB) and immunohistochemistry (13). Similarly, Zhou et al. identified eight BC patients with thyroid metastasis, mostly asymptomatic, and responsive to chemotherapy (14). These cases suggest that in patients with thyroid nodules, BC metastasis should be considered.

Since there have been clinical reports of an association between BC and thyroid neoplasms, several studies have suggested a potential link between BC and thyroid carcinoma, with shared genetic mutations being a key factor. Mutations in PTEN, associated with Cowden syndrome, increase the risk of both BC and thyroid neoplasms (15, 16). Inflammatory pathways like STAT3 may also contribute, as evidenced by elevated STAT3 activity in both BC and thyroid carcinoma patients (17, 18). Premalignant thyroid lesions, particularly nodules, are common in BC patients, increasing their risk of developing thyroid cancer (8, 19). Evidence from population studies also suggests a potential link. For example, Shi et al. found a higher incidence of thyroid nodules and dysfunction in BC patients, which may elevate the risk of malignant transformation (8). Also, a retrospective study identified shared genetic risk factors between BC and thyroid malignancies (20). Although this retrospective study is limited in establishing the temporal sequence between exposure and outcome, making it difficult to determine causality, it suggests that we can analyze the causal relationship from a genetic perspective. However, other studies have not found significant evidence supporting this association (21). While large-scale epidemiological studies or RCTs specifically addressing the BC-thyroid neoplasms relationship are limited (22, 23), so there is an urgent need for a “naturally occurring RCT” to objectively and rigorously investigate this association.

MR is a robust method for assessing causal relationships and is generally more reliable than traditional multivariable regression due to its ability to mitigate confounding and reverse causation, often referred to as a “naturally occurring RCT” (24–28). Liu et al. utilized MR to investigate the causal link between BC and malignant thyroid tumors, finding a significant impact of BC on thyroid carcinoma development (29). However, this study did not address benign thyroid neoplasms and the causal relationship was not strengthened by accounting for some common confounding factors by a MVMR analysis.

Due to shared molecular and genetic characteristics, MR is a valuable tool for investigating the causal relationship between BC and neoplasms of the thyroid gland from the perspective of susceptibility genes, providing valuable and deeper insights into the causal mechanisms underlying these associations. Our research extends UVMR and MVMR analyses to evaluate overall BC, which included Luminal A, Luminal B, HER2-positive, and TNBC, in relation to both malignant and benign thyroid neoplasms, enhancing the understanding of the genetic underpinnings of these diseases. In this “naturally occurring RCT”, we employed the OR to evaluate the relative likelihood of developing neoplasms of the thyroid gland in individuals with BC compared to those without BC, quantifying the increased risk associated with the presence of BC. These findings offer important implications for future research and clinical practice, emphasizing the need for continued investigation into the genetic links between BC and thyroid neoplasms.

## 2 Materials and methods

### 2.1 Study design

**Figure 1:**
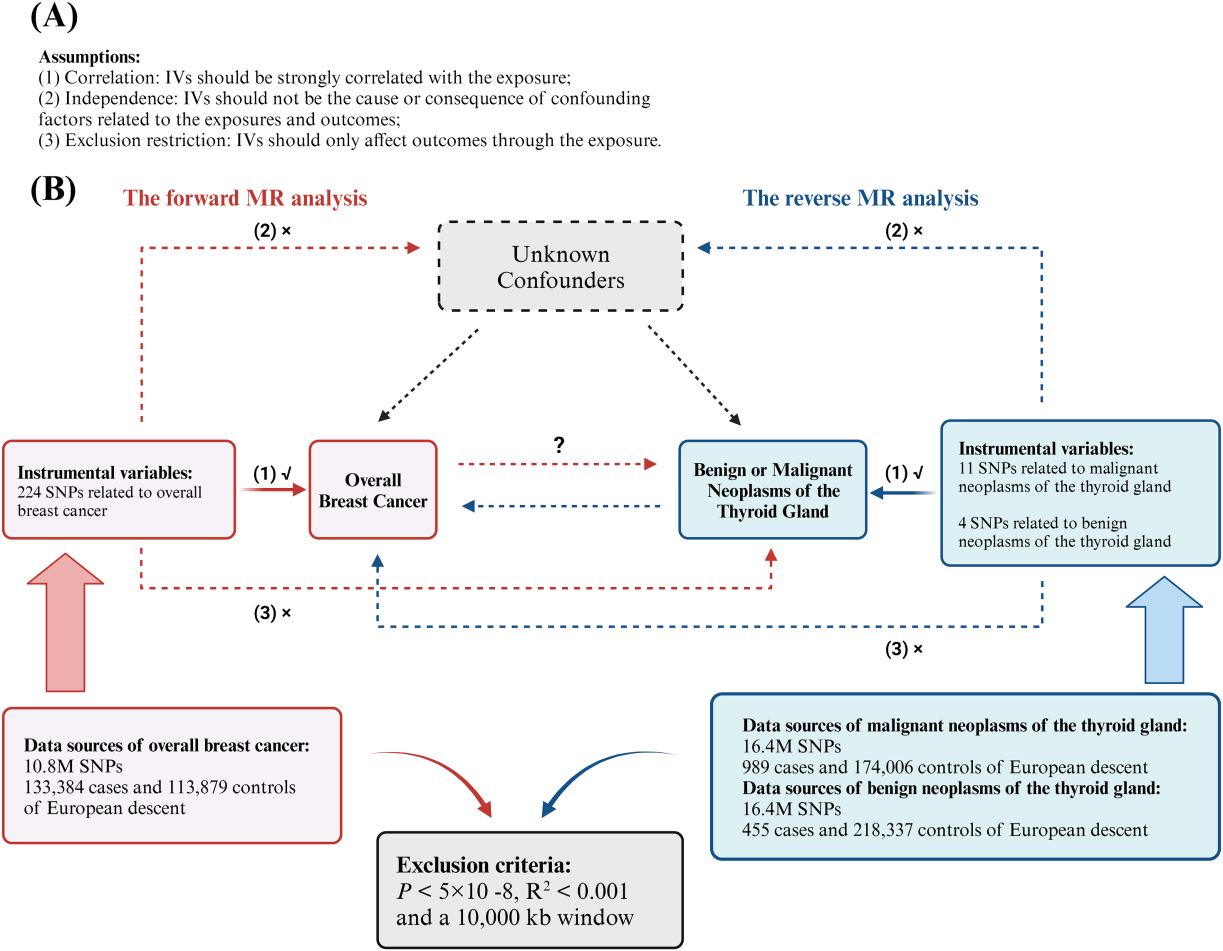
Analytical schematic diagram of the bidirectional univariable Mendelian randomization (MR) analysis. **(A)** MR analyses depend on three core assumptions; **(B)** Sketch of the study design. The red represented the forward MR analyses, with overall breast cancer as exposure and malignant or benign neoplasms of the thyroid gland respectively as the outcome. The blue represented the reverse MR analyses, with malignant or benign neoplasms of the thyroid gland respectively as exposure and overall breast cancer as the outcome.

**Figure 2:**
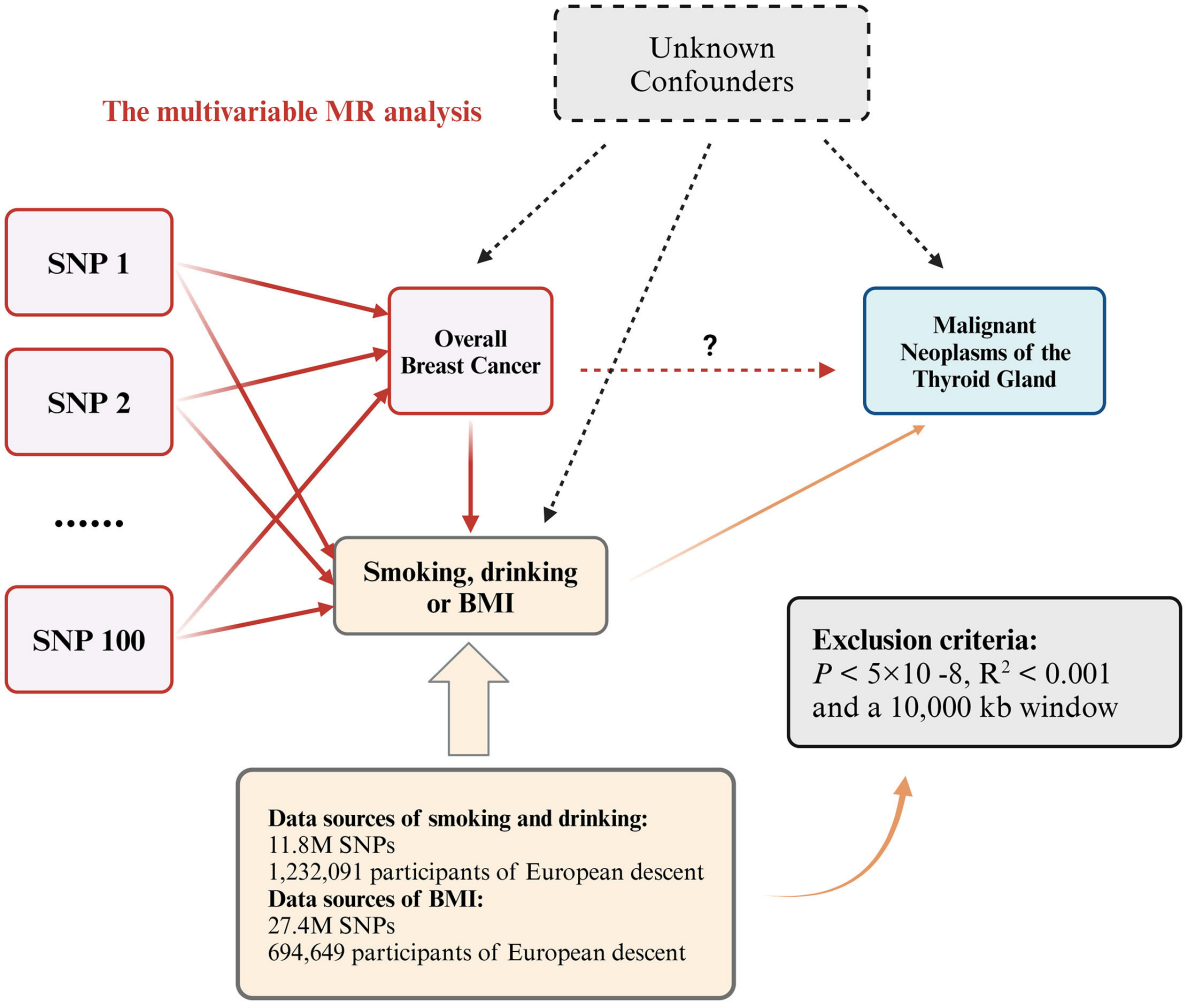
Multivariable MR allows an additional variable, besides the main exposure. We tested whether the three confounding factors of smoking, drinking and BMI could affect the causal relationship between overall breast cancer and malignant neoplasms of the thyroid gland. If the *P* value is still less than 0.05 after adjusting for confounding factors, it proves that the UVMR results are robust.

We performed a bidirectional two-sample MR analysis using instrumental variables (IVs). This analysis employed UVMR to evaluate the causal relationship between overall BC and various types of thyroid gland tumors, including both malignant and benign neoplasms. Initially, we used BC as the exposure and thyroid gland tumors as the outcome to investigate the forward association between these diseases. Subsequently, we assessed the reverse causal relationship by treating different types of thyroid neoplasms (both malignant and benign) as exposures and BC as the outcome, to determine the causal effects in the opposite direction (**Figure 1B**). To enhance our understanding and address potential confounders, we conducted a supplementary analysis using MVMR (**Figure 2**). This approach aimed to isolate the independent effects of BC and thyroid neoplasms while mitigating the impact of common confounding variables, thereby strengthening the results obtained from the UVMR analysis (30).

### 2.2 Data sources

#### 2.2.1 Breast cancer

In 2020, Zhang et al. conducted a study on identifying BC susceptibility loci using genome-wide association study (GWAS) methods (31). This research drew from multiple datasets, primarily involving European women from over 20 countries, utilizing data from the iCOGS and OncoArray platforms in 82 BCAC studies. These BCAC studies included large-scale population-based case-control studies, hospital-based case-control studies, and population-based cohort studies, all of which collected detailed information on clinical and pathological characteristics. The BC dataset represented overall BC, which encompassed all subtypes mentioned above, and included 133,384 individuals diagnosed with BC and 113,789 control individuals without BC (**Supplementary Table S1**).

#### 2.2.2 Neoplasms of the thyroid gland

We selected data on malignant and benign thyroid gland neoplasms from the FinnGen dataset. The malignant neoplasms of the thyroid gland dataset included 989 cases and 174,006 controls of European ancestry, with data on 16.4 million SNPs, excluding cases of other cancers. Similarly, the benign thyroid neoplasm dataset comprised 455 cases and 218,337 controls of European ancestry, also with data on 16.4 million SNPs. (**Supplementary Table S1**).

#### 2.2.3 Smoking and drinking

Liu et al. investigated the genetic factors influencing cigarette and alcohol consumption in the European population (32). This meta-analysis illuminated the complex interactions of these factors. The study encompassed a large cohort of 1.2 million Europeans, providing a comprehensive and insightful analysis (**Supplementary Table S1**).

#### 2.2.4 BMI

In 2019, a meta-analysis was conducted to explore the relationship between adiposity and genetic factors. Pulit et al. aimed to investigate the genetic factors associated with BMI and to identify novel loci that contribute to the distribution of adipose tissue *in vivo* (33). The study utilized BMI data from the UK Biobank (UKBB) dataset (https://www.ukbiobank.ac.uk/), a comprehensive repository of health-related information. This dataset, known for its extensively imputed genotype data, has become a valuable resource for genetic research (**Supplementary Table S1**).

### 2.3 Instrument selection

In MR studies, IVs must meet three key requirements: (1) they should be strongly correlated with the exposure (*correlation assumption*); (2) they should not be the cause or consequence of confounding factors related to the exposures and outcomes (*independence assumption*); and (3) they should only affect outcomes through the exposure (*exclusion restriction assumption*) (34) (**Figure 1A**). We identified SNPs that exhibited a strong correlation with the exposure variable following the selection criteria: *P* < 5×10^-8^. To eliminate linkage disequilibrium (LD), we applied a strict cutoff of R^2^ < 0.001 and a 10,000 kb window criterion. To fulfill assumption (1), SNPs that exhibited a strong association with the outcome variable were excluded. Subsequently, we harmonized the effects of SNPs on both exposure and outcome, ensuring that β values were annotated with the same allele. After these procedures, the remaining SNPs were deemed suitable for MR analysis.

A significant issue in MR studies is weak instrument bias. Weak IVs refer to genetic variations with lower explanatory power of the exposure, indicating that while the IVs affect the exposure, the strength of this association is not very high. The F statistic is an effective estimator for evaluating weak IV effects. When F is greater than 10, it indicates a relatively low risk of weak instrument bias in MR analysis (35). The calculation formula for the F statistic is:


$$F=\frac{R^{2}\times \left(N-k-1\right)}{k\times {\left(1-R\right)}^{2}}$$

where N represents the number of samples exposed in GWAS, and k stands for the number of IVs. R² represents the degree of exposure explained by the IVs and is the coefficient of determination of the regression equation (36). R² was evaluated from the correlation between the effect allele frequency (EAF) and the SNP-exposure association (β) (37), calculated using the following formula:


$$R^{2}=2\times EAF\times \left(1-EAF\right)\times \beta^{2}$$

### 2.4 Statistical analyses

#### 2.4.1 Forward UVMR and sensitivity analysis

Our primary assessment of the causal effect between overall BC and neoplasms of the thyroid gland is derived from the results of the inverse-variance-weighted (IVW) method. The IVW approach provides the most accurate estimates when no horizontal pleiotropy exists among the SNPs (38). To further enhance the robustness of our findings, we employed secondary analyses, including the weighted median and MR-Egger regression methods, to corroborate the IVW results. The weighted median method is particularly robust in the presence of heterogeneity, as it excludes up to 50% of invalid IVs from the analysis (38). MR-Egger regression, on the other hand, is adept at detecting and accounting for horizontal pleiotropy, providing reliable estimates even when pleiotropy is present (39).

We subsequently conducted sensitivity analyses to evaluate the heterogeneity of genetic variants, the validity of the selected IVs, potential horizontal pleiotropy, and the impact of outliers on our results. The intercept from the MR-Egger regression was used to assess bias and horizontal pleiotropy caused by invalid IVs (39). Additionally, heterogeneity among the SNPs was indicated by a *P*-value < 0.05 from Cochran’s Q test (40). To further validate the robustness of our findings, we performed a leave-one-out (LOO) analysis. This approach involved sequentially removing individual SNPs from the MR analysis and reanalyzing the data to assess their influence on the overall results (39).

In this study, we set the type I error rate at 0.05 and calculated statistical power based on sample size, the R² of the IVs, effect size, and the variance of both the exposure and outcome variables (**Figure 1B**).

#### 2.4.2 Assessment of the directionality of the causality

The observed forward causal relationship may be subject to bias from various factors. In addition to confounding, reverse causality could also contribute to bias. To address these potential sources of bias, we performed both reverse MR analysis and MVMR analysis to rule out any confounding effects and further validate the integrity of our findings.

#### 2.4.3 Reverse UVMR and sensitivity analysis

We conducted a reverse MR analysis to investigate the potential reverse causal relationship between neoplasms of the thyroid gland (exposure) and overall BC (outcome). Using the same criteria for IV selection, we applied three MR methods including IVW, weighted median, and MR-Egger, to explore the potential causal link between the two conditions. After conducting the analysis, we used Cochran’s Q test (*P* < 0.05) to assess heterogeneity. The MR-Egger intercept was employed to examine horizontal pleiotropy and bias caused by invalid IVs. Finally, we performed a LOO analysis to confirm the robustness of our findings (**Figure 1B**).

#### 2.4.4 Confounding analysis and MVMR

Despite conducting multiple sensitivity analyses to assess horizontal pleiotropy in the UVMR results, it is important to remain cautious about the potential association of SNPs with confounding factors. MVMR is an extension of the MR method that allows for the inclusion of SNPs associations with multiple phenotypes within a single analysis, enabling the estimation of the direct effect of each phenotype on the outcome (41). To account for potential confounding factors and mitigate the impact of horizontal pleiotropy, we conducted a MVMR analysis as an additional approach. Previous studies have suggested that factors such as smoking, alcohol consumption and BMI may influence the risk of both overall BC and malignant neoplasms of the thyroid gland (42). Therefore, we included variables as smoking status, alcohol consumption, and BMI in our analysis to determine whether they affect the causal relationship between BC and malignant neoplasms of the thyroid gland. This approach aimed to enhance the robustness and credibility of the results obtained from the UVMR analysis (**Figure 2**).

All the analyses in this study were conducted using the RStudio (Build 524) and R (Version 4.3.2) software packages which employ R packages such as “TwosampleMR”, “MRInstruments” and “MVMR”. Since we used available summary data from the public database, there was no need for ethical approval.

### 2.5 Assessment of MR study quality

#### 2.5.1 Instrument strength

To ensure that the IVs are sufficiently strong, the F-statistic should exceed 10. This threshold indicates that the IVs have a robust association with the exposure variable.

#### 2.5.2 Correlation with exposure

The *P*-value for the SNP-exposure association should be highly significant, typically less than 5×10^-8^, to ensure a strong correlation between the SNPs and the exposure variable. When the threshold of *P* = 5×10^-8^ fails to identify sufficient relevant SNPs or results in an excessively small number of SNPs, the threshold may be relaxed to *P* = 5×10^-6^.

#### 2.5.3 LD threshold

To minimize LD, SNPs should be independent of one another with an R² value less than 0.001. This criterion ensures that the SNPs are not in high LD, thereby reducing potential bias in the analysis.

#### 2.5.4 Interpretation of UVMR and MVMR analyses

In this study, OR was employed to assess whether exposure to BC is associated with an increased likelihood of neoplasms of the thyroid gland. OR quantifies the relative odds of an event occurring in an exposed group compared to a non-exposed group, offering a measure of the association’s strength and direction between an exposure and an outcome. The strength of the causality between the exposure and the outcome was evaluated using the association strength criteria proposed by Monson in 1980 (**Supplementary Table S2**). A weak odds ratio refers to an odds ratio that is close to 1. Specifically, OR value close to 1 (0.9~1.0, 1.0~1.1) indicates nearly no association between exposure and outcome (43), an OR value ranging from 1.2 to 1.5 signifies a weak correlation (44), an OR value ranging from 1.5 to 3.0 refers to a moderate association (45), whereas an OR approaching 0 or positive infinity suggests an extremely strong association. A weak OR refers to values that are close to 1. For instance, in a MR study conducted by Zhu et al., which investigated the causal relationship between polyunsaturated fatty acids and Parkinson’s disease, the OR for arachidonic acid in relation to Parkinson’s disease was found to be 1.05. This value is notably close to 1, and the authors therefore categorized it as a weak OR (46). However, due to the large sample sizes typically employed in MR studies, it is entirely possible to observe a statistically significant *P*-value while obtaining a weak OR due to a small effect size between a risk factor and a disease that truly existed (45).

In assessing the causal relationship between the exposure and outcome, the primary analysis is conducted using the IVW method. A significance level of *P* < 0.05 in the IVW analysis provides initial evidence of a causal association. The robustness of this causal relationship is further supported if both the weighted median and MR-Egger methods also yield *P*-values less than 0.05 and produce consistent OR values with those obtained from the IVW analysis. In general, a causal relationship can only be considered robust when the ORs obtained from the IVW, MR-Egger, and weighted median methods are consistent, and the corresponding *P*-values are statistically significant across all methods. Normally, the MR-Egger intercept *P*-value and Cochran’s Q test *P*-value being less than 0.05 suggest that there is potential significant horizontal pleiotropy between SNPs. However, such a situation may hold under certain conditions. *P* < 0.05, suggesting heterogeneity among SNPs, the primary results from the IVW and weighted median methods, along with the LOO analysis, remain consistent. This suggests that the horizontal pleiotropy is likely distributed across the SNPs rather than unduly affecting the overall causal estimate. Therefore, the selection of SNPs is considered reliable, and the causal relationship determined by the UVMR remains robust. To further strengthen and validate the UVMR findings, we conducted a MVMR analysis. This additional analysis aims to confirm the robustness of the causal relationship and address potential issues of horizontal pleiotropy. If, after adjusting for confounding factors in the MVMR analysis, the *P*-value for the causal relationship between overall BC and malignant neoplasms of the thyroid gland remains less than 0.05, this indicates that the causal association is not affected by confounding factors, consistent with the results from the UVMR.

In this study, all data analyses were performed through three independent repetitions and validated by experts from three different institutions: a statistician with 14 years of experience, another statistician with 16 years of experience, and an epidemiologist with 33 years of expertise. This thorough validation process ensured the robustness and accuracy of the results. Additionally, all experts involved had no conflicts of interest.

## 3 Results

### 3.1 UVMR

Based on the IV selection criteria, we identified 224 SNPs associated with BC (**Supplementary Table S3**), 11 SNPs linked to malignant thyroid neoplasms (**Supplementary Table S4**), and 4 SNPs related to benign thyroid neoplasms (**Supplementary Table S5**). All selected IVs demonstrated an F-statistic well above 10, indicating that instrument bias is unlikely among the chosen IVs.

#### 3.1.1 The forward MR analysis

##### 3.1.1.1 The causal effect of overall BC on malignant neoplasms of the thyroid gland

As shown in **Table 1**, the IVW method provides preliminary evidence of a positive, causal relationship between overall BC and malignant neoplasms of the thyroid gland from a genetic correlation perspective (OR and 95% CI: 1.291, 1.143-1.458, *P* = 3.90×10^-5^). Similarly, results from the MR-Egger (OR and 95% CI: 1.617, 1.258-2.079, *P* = 2.40×10^-4^) and weighted median (OR and 95% CI: 1.175, 1.005-1.374, *P* = 4.30×10^-2^) methods align with those of the IVW method, further confirming the causal relationship between these two conditions. The results from the IVW method indicate that the risk of developing malignant neoplasms of the thyroid gland in individuals with overall BC is about 1.3 times higher compared to those without overall BC. According to Monson’s criteria for assessing the strength of association, the causal relationship between overall BC and malignant neoplasms of the thyroid gland is evident (*P* < 0.05). Although **Table 2** shows evidence of horizontal pleiotropy and heterogeneity among SNPs in the MR-Egger regression and Cochran’s Q test (*P* < 5×10^-2^), the LOO analysis demonstrates that the causal effect estimates remain largely consistent when each IV is sequentially removed (**Supplementary Table S6**). This indicates that the selected IVs are robust and the results are reliable.

**Table 1:** Forward and reverse UVMR and sensitivity results for causality between overall BC and neoplasms of the thyroid gland.

Exposure	Outcome	Result	IVW	MR-Egger	Weighted Median
*The forward MR analyses*					
Overall breast cancer	Malignant neoplasms of the thyroid gland	OR (95% CI)	1.291 (1.143-1.458)	1.617 (1.258-2.079)	1.175 (1.005-1.374)
		*P*-value	3.90E-05	2.40E-04	4.30E-02
	Benign neoplasms of the thyroid gland	OR (95% CI)	1.052 (0.924-1.199)	1.038 (0.788-1.367)	1.190 (0.961-1.474)
		*P*-value	0.44	0.79	0.11
*The reverse MR analyses*					
Malignant neoplasms of the thyroid gland	Overall breast cancer	OR (95% CI)	1.091 (0.974-1.222)	2.004 (1.543-2.602)	1.000 (0.966-1.035)
		*P*-value	0.13	1.20E-03	1.00
Benign neoplasms of the thyroid gland		OR (95% CI)	0.985 (0.976-0.994)	0.942 (0.652-1.360)	0.983 (0.951-1.015)
		*P*-value	1.30E-03	0.78	0.28

**Table 2:** Horizontal pleiotropy and heterogeneity analyses.

Exposure	Outcome	MR-Egger regression		Cochran’s Q test	
		Intercept	*P*-value	Q	*P*-value
*The forward MR analyses*					
Overall breast cancer	Malignant neoplasms of the thyroid gland	-0.02	4.66E-02	361.95	7.87E-13
	Benign neoplasms of the thyroid gland	0.00	0.91	202.65	0.25
*The reverse MR analyses*					
Malignant neoplasms of the thyroid gland	Overall breast cancer	-0.14	2.24E-03	214.11	6.76E-42
Benign neoplasms of the thyroid gland		0.01	0.83	0.36	0.95

##### 3.1.1.2 The causal effect of overall BC on benign neoplasms of the thyroid gland

As shown in **Table 1**, the IVW method does not support a causal relationship between overall BC and benign neoplasms of the thyroid gland (OR and 95% CI: 1.052, 0.924-1.199, *P* = 0.44). Similarly, neither the MR-Egger method nor the weighted median method suggests a causal association between these two diseases (*P* > 5×10^-2^). The analysis presented in **Table 2** demonstrates that the *P*-value of the MR-Egger regression intercept and the *P*-value for Cochran’s Q test are both greater than 0.05, indicating the absence of horizontal pleiotropy and heterogeneity among SNPs. Furthermore, the LOO analysis, which sequentially removes each SNP, shows no significant changes in the results (**Supplementary Table S7**), further validating the reliability of the findings. Thus, there is no causal association between overall BC and benign neoplasms of the thyroid gland.

#### 3.1.2 The reverse MR analysis

##### 3.1.2.1 The causal effect of malignant neoplasms of the thyroid gland on overall BC

The results from the MR-Egger method suggest a potential causal relationship between malignant neoplasms of the thyroid gland and overall BC (OR and 95% CI: 2.004, 1.543-2.602, *P* = 1.20×10^-3^) (**Table 1**). However, the IVW method, which is considered the most robust, and the weighted median method, do not indicate such a causal relationship (*P* > 5×10^-2^), demonstrating that there is no causal effect of malignant neoplasms of the thyroid gland on overall BC. The analysis presented in **Table 2**, which includes MR-Egger regression and Cochran’s Q test, reveals significant horizontal pleiotropy and heterogeneity among SNPs (*P* < 5×10^-2^). However, the LOO analysis shown in **Supplementary Table S8** indicates that removing individual SNPs does not lead to significant changes in the results. This suggests that the observed pleiotropy and heterogeneity are distributed across the SNPs, and the selection of IVs remains reasonable and robust.

##### 3.1.2.2 The causal effect of benign neoplasms of the thyroid gland on overall BC

The IVW analysis presented in **Table 1** indicates a very slight negative, or, no causal relationship between benign neoplasms of the thyroid gland and overall BC from the perspective of genetic susceptibility (OR and 95% CI: 0.985, 0.976-0.994, *P* = 1.30×10^-3^). However, this finding is not supported by the MR-Egger or weighted median methods (*P* > 0.05), suggesting that the causal association between benign neoplasms of the thyroid gland and overall BC may not be reliable. This statistically significant OR value of only IVW method which is close to 1, may be attributed to the limited number of SNPs associated with neoplasms of the thyroid gland, which is only four (**Supplementary Table S5**). Insufficient number of SNPs can lead to unstable OR values (47). To increase the number of SNPs identified, we could consider relaxing the selection criteria by adjusting the significance threshold from *P* < 5×10^-8^ to *P* < 5×10^-5^. This adjustment would facilitate the identification of additional SNPs, but it would also increase the risk of false positives (48). Results in **Table 2** show that MR-Egger regression and Cochran’s Q test did not detect significant horizontal pleiotropy or heterogeneity among SNPs. The LOO analysis also did not identify any SNPs significantly affecting the results, further confirming the robustness and validity of the findings (**Supplementary Table S9**).

### 3.2 MVMR

In the MVMR analysis, we accounted for several potential confounders, including smoking status, drinking status, and BMI, to further investigate the independent effect of overall BC on malignant neoplasms of the thyroid gland.

#### 3.2.1 Adjusting for smoking status

**Figure 3:**
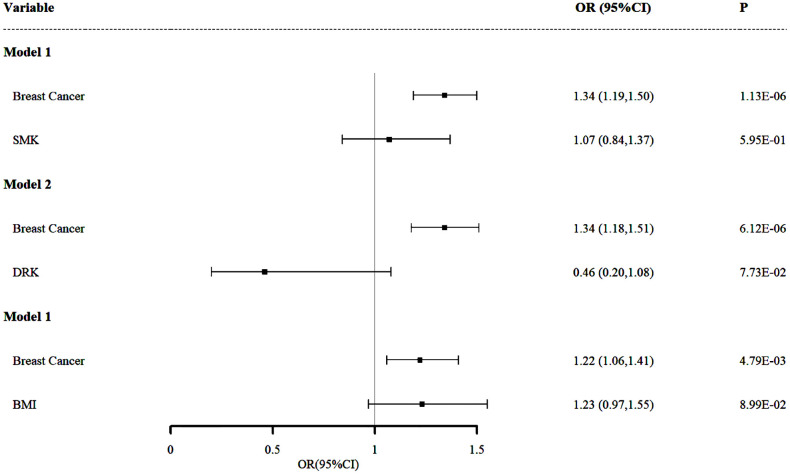
Independent effect of smoking, drinking and BMI on the risk of malignant neoplasms of the thyroid gland using multivariable Mendelian randomization analysis. Model 1: independent effect of overall BC on Malignant Neoplasms of the Thyroid Gland after adjusting smoking; Model 2: independent effect of overall BC on Malignant Neoplasms of the Thyroid Gland after adjusting drinking; Model 3: independent effect of overall BC on Malignant Neoplasms of the Thyroid Gland after adjusting BMI.

In Model 1 (**Figure 3**), we adjusted for smoking (SMK) as a potential confounder to examine the relationship between overall BC and malignant neoplasms of the thyroid gland. The results indicated that, even after accounting for smoking, there remains a significant positive association between overall BC and malignant neoplasms of the thyroid gland (OR and 95% CI: 1.34, 1.19-1.50, *P* = 1.13×10^-6^). This suggests that overall BC may be an independent risk factor for malignant neoplasms of the thyroid gland, with its effect not being significantly influenced by smoking behavior and individuals with overall BC have a 1.34-fold increased risk of developing malignant neoplasms of the thyroid gland compared to those without overall BC. This finding underscores the importance of overall BC as an exposure of malignant neoplasms of the thyroid gland and also suggests that smoking may not be a major explanatory factor for the association between these two cancers.

#### 3.2.2 Adjusting for drinking status

In Model 2 (**Figure 3**), we further examined the impact of drinking status (DRK) as a potential confounder on the relationship between overall BC and malignant neoplasms of the thyroid gland. The results demonstrated that even after accounting for drinking status, the significant positive association between overall BC and malignant neoplasms of the thyroid gland persisted (OR and 95% CI: 1.34, 1.18-1.51, *P* = 6.12×10^-6^). This finding is consistent with the results from Model 1, reinforcing the hypothesis that overall BC is an independent risk factor for malignant neoplasms of the thyroid gland, revealing that the presence of overall BC is associated with a 34% increased risk of developing malignant neoplasms of the thyroid gland. This finding suggests that while drinking might influence the risk of malignant neoplasms of the thyroid gland, it does not explain the strong association between these two diseases.

#### 3.2.3 Adjusting for BMI

In Model 3 (**Figure 3**), we adjusted for BMI, an important potential confounder, to assess its impact on the relationship between overall BC and malignant neoplasms of the thyroid gland. The results revealed that, even after accounting for BMI, the positive association between overall BC and malignant neoplasms of the thyroid gland remained significant (OR and 95% CI: 1.22, 1.06-1.41, *P* = 4.79×10^-3^), which indicates that the risk of developing malignant neoplasms of the thyroid gland is 1.22 times higher in individuals with overall BC compared to those without overall BC. Although this association is somewhat weaker than in the previous two models, it remains statistically significant and the OR value is greater than 1.2, further supporting the hypothesis that overall BC is an independent risk factor for malignant neoplasms of the thyroid gland.

## 4 Discussion

Our study aims to elucidate the causal relationship between overall BC and thyroid gland neoplasms, with a particular focus on malignant thyroid neoplasms. Previous research has indicated a potential association between these two conditions. However, their analysis overlooked benign thyroid lesions, and consequently, the robustness of the findings is somewhat compromised. The motivation for this research stems from the clinical observation that BC and thyroid malignancies often co-occur, prompting questions about underlying shared mechanisms. Additionally, this study is inspired by previous research highlighting genetic commonalities between these two diseases, which suggests that shared genetic factors might play a role in their co-occurrence. For example, in BC research, Tracy et al. identified that the upregulation of mitotically associated long noncoding RNA (MANCR) is associated with lower patient survival rates (49). Similarly, Lu et al. found the same pattern in thyroid cancer patients, suggesting that elevated MANCR expression may be a shared pathogenic factor between breast and thyroid cancers (50). By integrating these observations and inspirations, our research aims to further investigate and elucidate the causal relationship between BC and thyroid malignancies through the lens of genetic susceptibility. By leveraging genetic data and MR techniques, our study seeks to provide a clearer understanding of these associations and address gaps in current knowledge.

In this study, we employed MR to explore the causal relationship between BC and thyroid gland neoplasms. Our findings offer compelling evidence that BC is causally associated with malignant thyroid gland tumors, while no such relationship was observed for benign thyroid tumors. The reverse causal analyses, examining whether thyroid gland neoplasms could influence BC, also yielded no significant evidence of an effect. These results were further validated through MVMR analyses, which accounted for potential confounding factors and controlled for horizontal pleiotropy, thereby eliminating any potential influence that might not affect the causal relationship. This confirmed that the observed causal association between overall BC and malignant neoplasms of the thyroid gland aligns with our initial UVMR findings, indicating a robust and reliable relationship.

Our study’s findings align with the observations of Joseph et al., who conducted a meta-analysis revealing the risk of developing thyroid carcinoma after BC increases by 17% (51). This highlights the clinical relevance of investigating the relationship between BC and thyroid neoplasms. Building on the genetic similarities between BC and thyroid neoplasms, we further explored their broader biological connections, discussing the potential biological mechanisms. Previous research by Piek et al. identified common triggers such as estrogen signaling and environmental factors, which may underpin the observed association between these tumors (23). The role of estrogen and progesterone in both BC and thyroid carcinoma suggests a potential shared mechanism. Transcriptional cross-talk between thyroid hormones and sex hormones plays a significant role in cancer development and response to treatment (52). Breast tissue is the main target of estrogen and progesterone and is sensitive to these two hormones (53). Both types of neoplasms of the thyroid gland are highly sensitive to circulating estrogen, similar to breast tissue (54). Estrogen’s impact on thyroid carcinoma, which has been linked to increased risk in females, may help explain the higher incidence rates observed (55). Moreover, the concept of premalignant lesions, as discussed by Shi et al., provides insight into the potential pathway through which BC patients might develop secondary thyroid cancer. Although chemotherapy can reduce the incidence of thyroid nodules by affecting the hypothalamic-pituitary-thyroid axis, timely intervention remains crucial for preventing thyroid cancer (8). This underscores the importance of proactive management in BC patients to mitigate the risk of secondary malignancies. BC patients should actively engage in treatment, initiating therapy as soon as the condition is detected.

Despite these insights, our study has several limitations. Firstly, the generalizability of our findings is limited by the use of data exclusively from European populations. This constraint means that our results may not fully reflect the experiences of other demographic groups, potentially affecting their applicability to non-European populations. Future research should aim to include more diverse populations to enhance the generalizability of the findings. Secondly, BC is a heterogeneous disease with multiple subtypes. Our analysis focused on overall BC data, leaving the causal relationships between specific BC subtypes and thyroid gland neoplasms unexplored. Investigating these subtype-specific associations is crucial for a more comprehensive understanding of the link between BC and thyroid neoplasms. Additionally, while we have made efforts to ensure rigorous data collection, the possibility of misclassification and detection bias cannot be entirely excluded. Misclassification may occur if patients are inaccurately categorized, while detection bias might arise from varying likelihoods of detecting conditions across different groups. Despite using high-quality databases, any inherent misclassification or detection bias within these sources could influence our results. Addressing these issues in future studies could improve the accuracy and reliability of the findings.

In our MR analysis, we accounted for several common confounding factors. However, there remains uncertainty about the influence of other potential confounders that were not considered. This limitation underscores the need for more extensive research to identify and address additional confounding variables that might affect the observed relationship between BC and thyroid malignancies. Moreover, MR analysis relies on three critical assumptions: the *correlation assumption*, the *independence assumption*, and the *exclusion restriction assumption*. For our results to be credible, these assumptions must be met and we have made efforts to adhere to these assumptions. For readers to accept the validity of these results, they must acknowledge the three core assumptions underlying MR. It is also important to acknowledge that the SNPs we selected may exhibit potential horizontal pleiotropy. While this does not appear to affect our overall results, it remains a factor that should be considered when interpreting our findings.

In addition, according to Monson’s criteria, there are weak ORs or no associations (0.9~1.0, 1.0~1.1) in our study, although it does not affect the results. Several factors could contribute to this situation, and the limitation mentioned above that not all potential confounders could be fully accounted for and adjusted is one reason. Moreover, the slight heterogeneity among SNPs is also a contributing factor to the weak OR. Meanwhile, the insufficient number of SNPs of benign neoplasms of the thyroid gland can also lead to unreliable OR results (44, 45). The sample size may also contribute to the weak OR. However, it is possible for a large sample size to yield statistically significant *P*-values due to genuinely small effects. As highlighted by Zhu et al. in their research, the complex molecular mechanisms underlying diseases may result in weak OR values for causal relationships, even though a risk association between the conditions does indeed exist (43), which means the causal relationship under investigation itself exhibits a weak effect (45). This also reflects one of the inherent limitations of MR methods. While MR techniques offer valuable insights into causal relationships, they do not elucidate the underlying mechanisms driving cancer development. The mechanism of occurrence between these two diseases needs further exploration.

From the perspective of genetic susceptibility, we have utilized MR to reveal the causal relationship between overall BC and neoplasms of the thyroid gland. Moving forward, we plan to investigate the nature of shared susceptibility genes between these conditions. This next step will involve elucidating the mechanisms at the genetic and protein molecular levels, and further expanding our understanding to cellular and tissue levels to comprehensively uncover how these mechanisms influence cancer development and progression.

## Data Availability

The original contributions presented in the study are included in the article/**Supplementary Material**. Further inquiries can be directed to the corresponding authors.

## References

[1] Harbeck N Gnant M. Breast cancer. Lancet. (2017) 389:1134–50. doi: 10.1016/S0140-6736(16)31891-8 27865536

[2] Giaquinto AN Sung H Miller KD Kramer JL Newman LA Minihan A. Breast cancer statistics, 2022. CA Cancer J Clin. (2022) 72(6):524–41. doi: 10.3322/caac.21754 36190501

[3] Orrantia-Borunda E Anchondo-Nuñez P Acuña-Aguilar LE Gómez-Valles FO Ramírez-Valdespino CA. Subtypes of breast cancer. In: Mayrovitz HN, editor. Breast cancer. Exon Publications, Brisbane (AU (2022). 36122153

[4] Britt KL Cuzick J Phillips KA. Key steps for effective breast cancer prevention. Nat Rev Cancer. (2020) 20:417–36. doi: 10.1038/s41568-020-0266-x 32528185

[5] McDonald ES Clark AS Tchou J Zhang P Freedman GM. Clinical diagnosis and management of breast cancer. J Nucl Med. (2016) 57 Suppl 1:9S–16S. doi: 10.2967/jnumed.115.157834 26834110

[6] Keenan TE Tolaney SM. Role of immunotherapy in triple-negative breast cancer. J Natl Compr Canc Netw. (2020) 18:479–89. doi: 10.6004/jnccn.2020.7554 32259782

[7] Jereczek-Fossa BA Alterio D Jassem J Gibelli B Tradati N Orecchia R. Radiotherapy-induced thyroid disorders. Cancer Treat Rev. (2004) 30:369–84. doi: 10.1016/j.ctrv.2003.12.003 15145511

[8] Shi Y Li X Ran L Arshad B Li H Xu Z. Study on the status of thyroid function and thyroid nodules in chinese breast cancer patients. Oncotarget. (2017) 8(46):80820–5. doi: 10.18632/oncotarget.20542 PMC5655241 29113346

[9] Karaviti D Kani ER Karaviti E Gerontiti E Michalopoulou O Stefanaki K. Thyroid disorders induced by immune checkpoint inhibitors. Endocrine. (2024) 85(1):67–79. doi: 10.1007/s12020-024-03718-2 38345684 PMC11246261

[10] Baloch ZW Asa SL Barletta JA Ghossein RA Juhlin CC Jung CK. Overview of the 2022 WHO classification of thyroid neoplasms. Endocr Pathol. (2022) 33(1):27–63. doi: 10.1007/s12022-022-09707-3 35288841

[11] Xu F Liu B Chen XY Zhou EX Fan DF Ma Y. [Diagnosis and therapy of childhood thyroid carcinoma: clinical analysis of 12 cases]. Zhongguo Dang Dai Er Ke Za Zhi. (2009) 11(2):120–3. 19222949

[12] Sung H Ferlay J Siegel RL Laversanne M Soerjomataram I Jemal A. Global cancer statistics 2020: GLOBOCAN estimates of incidence and mortality worldwide for 36 cancers in 185 countries. CA Cancer J Clin. (2021) 71(3):209–49. doi: 10.3322/caac.21660 33538338

[13] Ramírez Stieben LA Vargas MC Polillo DC Lufft K Saldías PR Bedini I. Metastasis of breast cancer to the thyroid gland. Metástasis cáncer mama en glándula tiroides. Medicina (B Aires). (2024) 84:741–5. 39172574

[14] Zhou L Chen L Xu D Shao Q Guo Z Ge M. Breast cancer metastasis to thyroid: a retrospective analysis. Afr Health Sci. (2017) 17:1035–43. doi: 10.4314/ahs.v17i4.11 PMC5870294 29937874

[15] Bolf EL Sprague BL Carr FE. A linkage between thyroid and breast cancer: A common etiology? Cancer Epidemiol Biomarkers Prev. (2019) 28:643–9. doi: 10.1158/1055-9965.EPI-18-0877 30541751

[16] Liaw D Marsh DJ Li J Dahia PL Wang SI Zheng Z. Germline mutations of the PTEN gene in Cowden disease, an inherited breast and thyroid cancer syndrome. Nat Genet. (1997) 16(1):64–7. doi: 10.1038/ng0597-64 9140396

[17] Kim WG Park JW Willingham MC Cheng SY. Diet-induced obesity increases tumor growth and promotes anaplastic change in thyroid cancer in a mouse model. Endocrinology. (2013) 154:2936–47. doi: 10.1210/en.2013-1128 PMC3713208 23748362

[18] Lapeire L Hendrix A Lambein K Van Bockstal M Braems G Van Den Broecke R. Cancer-associated adipose tissue promotes breast cancer progression by paracrine oncostatin M and Jak/STAT3 signaling. Cancer Res. (2014) 74(23):6806–19. doi: 10.1158/0008-5472.CAN-14-0160 25252914

[19] Durante C Grani G Lamartina L Filetti S Mandel SJ Cooper DS. The diagnosis and management of thyroid nodules: A review. JAMA. (2018) 319:914–24. doi: 10.1001/jama.2018.0898 29509871

[20] Bakos B Kiss A Árvai K Szili B Deák-Kocsis B Tobiás B. Co-occurrence of thyroid and breast cancer is associated with an increased oncogenic SNP burden. BMC Cancer. (2021) 21(1):706. doi: 10.1186/s12885-021-08377-4 34130653 PMC8207626

[21] Pasqual E Schonfeld S Morton LM Villoing D Lee C Berrington de Gonzalez A. Association Between Radioactive Iodine Treatment for Pediatric and Young Adulthood Differentiated Thyroid Cancer and Risk of Second Primary Malignancies [published correction appears in J Clin Oncol. J Clin Oncol. (2022) 40(13):1439–49. doi: 10.1200/JCO.21.01841 PMC9061144 35044839

[22] Pan XF Ma YJ Tang Y Yu MM Wang H Fan YR. Breast cancer populations may have an increased prevalence of thyroglobulin antibody and thyroid peroxidase antibody: a systematic review and meta-analysis. Breast Cancer. (2020) 27:828–36. doi: 10.1007/s12282-020-01078-z 32279180

[23] Piek MW de Boer JP van Duijnhoven F van der Wal JE Vriens M van Leeuwaarde RS. The co-occurrence of both breast- and differentiated thyroid cancer: incidence, association and clinical implications for daily practice. BMC Cancer. (2022) 22(1):1018. doi: 10.1186/s12885-022-10069-6 36163009 PMC9511724

[24] Burgess S Davey Smith G Davies NM Dudbridge F Gill D Glymour MM. Guidelines for performing Mendelian randomization investigations: update for summer 2023. Wellcome Open Res. (2023) 4:186. doi: 10.12688/wellcomeopenres.15555.3 32760811 PMC7384151

[25] Bowden J Davey Smith G Burgess S. Mendelian randomization with invalid instruments: effect estimation and bias detection through Egger regression. Int J Epidemiol. (2015) 44:512–25. doi: 10.1093/ije/dyv080 PMC4469799 26050253

[26] Skrivankova VW Richmond RC Woolf BAR Davies NM Swanson SA VanderWeele TJ. Strengthening the reporting of observational studies in epidemiology using mendelian randomisation (STROBE-MR): explanation and elaboration. BMJ. (2021) 375:n2233. doi: 10.1136/bmj.n2233 34702754 PMC8546498

[27] Skrivankova VW Richmond RC Woolf BAR Yarmolinsky J Davies NM Swanson SA. Strengthening the reporting of observational studies in epidemiology using mendelian randomization: the STROBE-MR statement. JAMA. (2021) 326:1614–21. doi: 10.1001/jama.2021.18236 34698778

[28] Richmond RC Davey Smith G. Mendelian randomization: concepts and scope. Cold Spring Harb Perspect Med. (2022) 12:a040501. doi: 10.1101/cshperspect.a040501 34426474 PMC8725623

[29] Liu J Liang L. The association between thyroid and breast cancers: a bidirectional mendelian randomization study. Front Endocrinol (Lausanne). (2023) 14:1185497. doi: 10.3389/fendo.2023.1185497 37955011 PMC10634417

[30] Schooling CM Lopez PM Yang Z Zhao JV Au Yeung SL Huang JV. Use of multivariable mendelian randomization to address biases due to competing risk before recruitment. Front Genet. (2021) 11:610852. doi: 10.3389/fgene.2020.610852 33519914 PMC7845663

[31] Zhang H Ahearn TU Lecarpentier J Barnes D Beesley J Qi G. Genome-wide association study identifies 32 novel breast cancer susceptibility loci from overall and subtype-specific analyses. Nat Genet. (2020) 52(6):572–81. doi: 10.1038/s41588-020-0609-2 PMC7808397 32424353

[32] Liu M Jiang Y Wedow R Li Y Brazel DM Chen F. Association studies of up to 1.2 million individuals yield new insights into the genetic etiology of tobacco and alcohol use. Nat Genet. (2019) 51(2):237–44. doi: 10.1038/s41588-018-0307-5 PMC6358542 30643251

[33] Pulit SL Stoneman C Morris AP Wood AR Glastonbury CA Tyrrell J. Meta-analysis of genome-wide association studies for body fat distribution in 694 649 individuals of European ancestry. Hum Mol Genet. (2019) 28(1):166–74. doi: 10.1093/hmg/ddy327 PMC6298238 30239722

[34] Lawlor DA. Commentary: Two-sample Mendelian randomization: opportunities and challenges. Int J Epidemiol. (2016) 45:908–15. doi: 10.1093/ije/dyw127 PMC5005949 27427429

[35] Palmer TM Lawlor DA Harbord RM Sheehan NA Tobias JH Timpson NJ. Using multiple genetic variants as instrumental variables for modifiable risk factors. Stat Methods Med Res. (2012) 21(3):223–42. doi: 10.1177/0962280210394459 PMC3917707 21216802

[36] Burgess S Thompson SG CRP CHD Genetics Collaboration. Avoiding bias from weak instruments in Mendelian randomization studies. Int J Epidemiol. (2011) 40:755–64. doi: 10.1093/ije/dyr036 21414999

[37] Meddens SFW de Vlaming R Bowers P Burik CAP Linnér RK Lee C. Genomic analysis of diet composition finds novel loci and associations with health and lifestyle. Mol Psychiatry. (2021) 26(6):2056–69. doi: 10.1038/s41380-020-0697-5 PMC7767645 32393786

[38] Pierce BL Burgess S. Efficient design for Mendelian randomization studies: subsample and 2-sample instrumental variable estimators. Am J Epidemiol. (2013) 178:1177–84. doi: 10.1093/aje/kwt084 PMC3783091 23863760

[39] Burgess S Thompson SG. Interpreting findings from Mendelian randomization using the MR-Egger method. Eur J Epidemiol. (2017) 32:377–89. doi: 10.1007/s10654-017-0255-x PMC5506233 28527048

[40] Cohen JF Chalumeau M Cohen R Korevaar DA Khoshnood B Bossuyt PMM. Cochran’s Q test was useful to assess heterogeneity in likelihood ratios in studies of diagnostic accuracy. J Clin Epidemiol. (2015) 68:299–306. doi: 10.1016/j.jclinepi.2014.09.005 25441698

[41] Sanderson E. Multivariable mendelian randomization and mediation. Cold Spring Harb Perspect Med. (2021) 11::a038984. doi: 10.1101/cshperspect.a038984 32341063 PMC7849347

[42] Bowden J Del Greco MF Minelli C Zhao Q Lawlor DA Sheehan NA. Improving the accuracy of two-sample summary-data Mendelian randomization: moving beyond the NOME assumption. Int J Epidemiol. (2019) 48(3):728–42. doi: 10.1093/ije/dyy258 PMC6659376 30561657

[43] Haddock CK Rindskopf D Shadish WR. Using odds ratios as effect sizes for meta-analysis of dichotomous data: a primer on methods and issues. psychol Methods. (1998) 3.3:339. doi: 10.1037/1082-989X.3.3.339

[44] Grimes DA Schulz KF. Making sense of odds and odds ratios. Obstet Gynecol. (2008) 111:423–6. doi: 10.1097/01.AOG.0000297304.32187.5d 18238982

[45] Chen H Cohen P Chen S. How big is a big odds ratio? Interpreting the magnitudes of odds ratios in epidemiological studies. Commun Stat - Simulation Comput. (2010) 39:860–4. doi: 10.1080/03610911003650383

[46] Zhu X Huang S Kang W Chen P Liu J. Associations between polyunsaturated fatty acid concentrations and Parkinson's disease: A two-sample Mendelian randomization study. Front Aging Neurosci. (2023) 15:1123239. doi: 10.3389/fnagi.2023.1123239 36909950 PMC9992541

[47] Johnson AD Handsaker RE Pulit SL Nizzari MM O'Donnell CJ de Bakker PI. SNAP: a web-based tool for identification and annotation of proxy SNPs using HapMap. Bioinformatics. (2008) 24:2938–9. doi: 10.1093/bioinformatics/btn564 PMC2720775 18974171

[48] Tachmazidou I Süveges D Min JL Ritchie GRS Steinberg J Walter K. Whole-genome sequencing coupled to imputation discovers genetic signals for anthropometric traits. Am J Hum Genet. (2017) 100(6):865–84. doi: 10.1016/j.ajhg.2017.04.014 PMC5473732 28552196

[49] Tracy KM Tye CE Ghule PN Malaby HLH Stumpff J Stein JL. Mitotically-associated lncRNA (MANCR) affects genomic stability and cell division in aggressive breast cancer. Mol Cancer Res. (2018) 16(4):587–98. doi: 10.1158/1541-7786.MCR-17-0548 PMC5882506 29378907

[50] Lu W Xu Y Xu J Wang Z Ye G. Identification of differential expressed lncRNAs in human thyroid cancer by a genome-wide analyses. Cancer Med. (2018) 7:3935–44. doi: 10.1002/cam4.1627 PMC6089163 29923329

[51] Joseph KR Edirimanne S Eslick GD. The association between breast cancer and thyroid cancer: a meta-analysis. Breast Cancer Res Treat. (2015) 152:173–81. doi: 10.1007/s10549-015-3456-6 26058757

[52] Halada S Casado-Medrano V Baran JA Lee J Chinmay P Bauer AJ. Hormonal crosstalk between thyroid and breast cancer. Endocrinology. (2022) 163(7):bqac075. doi: 10.1210/endocr/bqac075 35587175 PMC9653009

[53] Draper CF Duisters K Weger B Chakrabarti A Harms AC Brennan L. Menstrual cycle rhythmicity: metabolic patterns in healthy women. Sci Rep. (2018) 8(1):14568. doi: 10.1038/s41598-018-32647-0 30275458 PMC6167362

[54] Santin AP Furlanetto TW. Role of estrogen in thyroid function and growth regulation. J Thyroid Res. (2011) 2011:875125. doi: 10.4061/2011/875125 21687614 PMC3113168

[55] Moleti M Sturniolo G Di Mauro M Russo M Vermiglio F. Female reproductive factors and differentiated thyroid cancer. Front Endocrinol (Lausanne). (2017) 8:111. doi: 10.3389/fendo.2017.00111 28588554 PMC5440523

