# Supplementary material for: Breast cancer and neoplasms of the thyroid gland: a bidirectional two-sample Mendelian randomization study
Source: Front Oncol. 2024 Oct 14;14:1422009. doi: 10.3389/fonc.2024.1422009 (PMC11513776; doi:10.3389/fonc.2024.1422009)
Supplement: Supplementary file 1 [file Table1.docx]

**Supplementary materials**

**Table S1. Description of the data sources for the phenotype employed.**

| **Phenotype** | **Data source** | **Total sample size** | **Population** | **SNPs** |  |
| --- | --- | --- | --- | --- | --- |
| Overall breast cancer | Zhang, Haoyu et al. “Genome-wide association study identifies 32 novel breast cancer susceptibility loci from overall and subtype-specific analyses.” Nature genetics vol. 52,6 (2020): 572-581. doi:10.1038/s41588-020-0609-2 | 133,384 cases 114,789 controls | European | 10.8 M |  |
| Malignant neoplasms of the thyroid gland | <https://storage.googleapis.com/finngen-public-data-r9/summary_stats/finngen_R9_C3_THYROID_GLAND_EXALLC.gz> | 989 cases 174,006 controls | European | 16.4 M |  |
| Benign neoplasms of the thyroid gland | <https://storage.googleapis.com/finngen-public-data-r9/summary_stats/finngen_R9_CD2_BENIGN_THYROID.gz> | 455 cases 218,337 controls | European | 16.4M |  |
|  |  |  |  |  |  |
| Smoking, drinking | Liu M, Jiang Y, Wedow R. et al. Association studies of up to 1.2 million individuals yield new insights into the genetic etiology of tobacco and alcohol use. Nat Genet. 2019 Feb;51(2):237-244. | 1,232,091 | European | 11.8M |  |
| BMI | Pulit SL, Stoneman C, Morris AP et al. Meta-analysis of genome-wide association studies for body fat distribution in 694 649 individuals of European ancestry. Hum Mol Genet. 2019 Jan 1;28(1):166-174. | 694, 649 | European | 27.4M |  |

SNPs: single nucleotide polymorphisms

**Table S2. RR (OR) with correlation strength**

| **RR (OR)** | | **correlation strength** |
| --- | --- | --- |
| **negative (<1)** | **positive (>1)** |  |
| 0.9~1.0 | 1.0~1.1 | No association |
| 0.7~0.8 | 1.2~1.4 | Weak |
| 0.4~0.6 | 1.5~2.9 | Moderate |
| 0.1~0.3 | 3.0~9.9 | Strong |
| <0.1 | 10~ | Extremely strong |
| **(Monson RA, 1980)** | | |

RR: relative risk

OR: odds ratio

**Table S3. Characteristics of GWAS-identified Overall Breast Cancer-associated genetic instruments.**

| **SNP** | **Chromosome** | **Position** | **Effect allele** | **Other allele** | **Effect allele frequency** | **Beta** | **Standard Error** | ***P*-value** | ***F*-statistic** |
| --- | --- | --- | --- | --- | --- | --- | --- | --- | --- |
| rs2506885 | 1 | 10581051 | T | A | 0.3512 | -0.0629 | 0.0066 | 1.88E-21 | 1254.3 |
| rs2992756 | 1 | 18807339 | C | T | 0.5207 | -0.0517 | 0.0061 | 2.67E-17 | 926.8 |
| rs4233486 | 1 | 41380440 | T | C | 0.6700 | 0.0363 | 0.0066 | 3.81E-08 | 404.0 |
| rs79724016 | 1 | 42137311 | G | T | 0.0307 | -0.1030 | 0.0172 | 2.10E-09 | 438.6 |
| rs1707302 | 1 | 46600917 | G | A | 0.6732 | 0.0371 | 0.0064 | 5.50E-09 | 421.2 |
| rs11583393 | 1 | 88429024 | A | C | 0.2460 | -0.0408 | 0.0070 | 4.86E-09 | 428.4 |
| rs7513707 | 1 | 114445880 | A | G | 0.1744 | 0.0554 | 0.0080 | 3.54E-12 | 614.4 |
| rs7529522 | 1 | 118230221 | C | T | 0.2416 | 0.0458 | 0.0072 | 2.45E-10 | 534.1 |
| rs637868 | 1 | 120257110 | C | T | 0.5404 | 0.0356 | 0.0061 | 5.89E-09 | 436.4 |
| rs372562666 | 1 | 120561314 | G | A | 0.2643 | 0.0954 | 0.0089 | 1.15E-26 | 2468.0 |
| rs201770099 | 1 | 121257040 | A | G | 0.4035 | 0.0645 | 0.0095 | 1.25E-11 | 1392.4 |
| rs11249433 | 1 | 121280613 | G | A | 0.4232 | 0.0998 | 0.0062 | 1.21E-57 | 3397.3 |
| rs36107432 | 1 | 145604791 | T | G | 0.3322 | -0.0395 | 0.0065 | 1.06E-09 | 482.1 |
| rs11205303 | 1 | 149906413 | C | T | 0.4105 | 0.0504 | 0.0062 | 5.07E-16 | 853.9 |
| rs12091730 | 1 | 155556971 | A | G | 0.2419 | 0.0499 | 0.0071 | 1.83E-12 | 634.5 |
| rs11264454 | 1 | 156153043 | G | A | 0.4385 | 0.0357 | 0.0063 | 1.71E-08 | 435.3 |
| rs35383942 | 1 | 201437832 | T | C | 0.0600 | 0.1046 | 0.0132 | 2.77E-15 | 858.6 |
| rs59867004 | 1 | 203801249 | C | T | 0.2798 | 0.0477 | 0.0067 | 1.35E-12 | 638.3 |
| rs11117758 | 1 | 217220574 | A | G | 0.2006 | -0.0434 | 0.0074 | 4.28E-09 | 419.3 |
| rs72755295 | 1 | 242034263 | G | A | 0.0360 | 0.1220 | 0.0172 | 1.38E-12 | 719.3 |
| rs113577745 | 2 | 10135681 | G | C | 0.1029 | 0.0619 | 0.0099 | 3.95E-10 | 491.5 |
| rs11684853 | 2 | 19310918 | T | G | 0.5504 | -0.0446 | 0.0060 | 1.29E-13 | 684.5 |
| rs2028195 | 2 | 25398953 | T | G | 0.2406 | 0.0446 | 0.0072 | 6.62E-10 | 505.5 |
| rs9712235 | 2 | 67881757 | A | G | 0.7359 | -0.0379 | 0.0069 | 4.78E-08 | 387.5 |
| rs4602255 | 2 | 69392128 | A | G | 0.4532 | 0.0361 | 0.0060 | 1.95E-09 | 449.6 |
| rs11903787 | 2 | 121088182 | A | G | 0.2331 | -0.0428 | 0.0073 | 3.81E-09 | 456.2 |
| rs4076654 | 2 | 121155824 | T | A | 0.3519 | -0.0476 | 0.0064 | 6.34E-14 | 719.8 |
| rs4849879 | 2 | 121239256 | G | C | 0.8956 | 0.0958 | 0.0097 | 3.18E-23 | 1195.2 |
| rs11680449 | 2 | 172369881 | A | G | 0.2721 | -0.0409 | 0.0067 | 9.14E-10 | 461.0 |
| rs2016394 | 2 | 172972971 | A | G | 0.4653 | -0.0406 | 0.0060 | 1.27E-11 | 569.7 |
| rs2010610 | 2 | 174210908 | G | C | 0.8509 | 0.0515 | 0.0083 | 5.06E-10 | 468.4 |
| rs13002632 | 2 | 201954867 | A | G | 0.0204 | 0.1313 | 0.0235 | 2.17E-08 | 479.2 |
| rs3769821 | 2 | 202123430 | T | C | 0.6568 | -0.0561 | 0.0063 | 6.09E-19 | 988.4 |
| rs2141818 | 2 | 217855058 | T | C | 0.0339 | 0.1023 | 0.0177 | 7.11E-09 | 477.0 |
| rs4442975 | 2 | 217920769 | T | G | 0.4714 | -0.1323 | 0.0059 | 1.03E-109 | 6110.3 |
| rs34005590 | 2 | 217963060 | A | C | 0.0410 | -0.1997 | 0.0147 | 6.25E-42 | 2185.5 |
| rs11693806 | 2 | 218292158 | G | C | 0.7108 | -0.0731 | 0.0068 | 2.75E-27 | 1531.0 |
| rs6436017 | 2 | 218723455 | G | A | 0.5050 | 0.0394 | 0.0061 | 1.16E-10 | 540.6 |
| rs12479355 | 2 | 227226952 | G | A | 0.2003 | -0.0431 | 0.0074 | 5.60E-09 | 414.2 |
| rs6762558 | 3 | 4742251 | G | A | 0.4107 | 0.0549 | 0.0061 | 3.73E-19 | 1015.1 |
| rs1375631 | 3 | 16778867 | G | A | 0.5054 | 0.0346 | 0.0060 | 6.79E-09 | 416.4 |
| rs552647 | 3 | 27353716 | A | C | 0.5489 | 0.1042 | 0.0060 | 1.86E-67 | 3755.9 |
| rs12493607 | 3 | 30682939 | C | G | 0.3568 | 0.0477 | 0.0063 | 2.72E-14 | 727.6 |
| rs56387622 | 3 | 46888198 | C | T | 0.0923 | -0.0927 | 0.0102 | 9.68E-20 | 1003.1 |
| rs2886671 | 3 | 59373745 | T | C | 0.4129 | -0.0353 | 0.0064 | 4.25E-08 | 419.4 |
| rs3821902 | 3 | 63941697 | G | T | 0.1409 | 0.0621 | 0.0086 | 6.98E-13 | 648.2 |
| rs6805189 | 3 | 71532113 | C | T | 0.4631 | -0.0379 | 0.0060 | 2.68E-10 | 496.2 |
| rs13066793 | 3 | 87037543 | G | A | 0.0882 | -0.0731 | 0.0109 | 1.71E-11 | 597.6 |
| rs9833888 | 3 | 99723580 | T | G | 0.2348 | 0.0466 | 0.0071 | 5.81E-11 | 542.7 |
| rs6440006 | 3 | 141142691 | A | G | 0.4605 | 0.0481 | 0.0060 | 1.59E-15 | 800.9 |
| rs1430411 | 3 | 156525396 | A | T | 0.6241 | 0.0339 | 0.0061 | 3.60E-08 | 374.0 |
| rs58058861 | 3 | 172285237 | A | G | 0.2261 | 0.0456 | 0.0072 | 2.40E-10 | 506.8 |
| rs6815814 | 4 | 38816338 | C | A | 0.2558 | 0.0512 | 0.0070 | 2.56E-13 | 694.4 |
| rs6854739 | 4 | 84367759 | T | A | 0.4882 | -0.0419 | 0.0060 | 2.39E-12 | 608.9 |
| rs10022462 | 4 | 89243818 | T | C | 0.4458 | 0.0362 | 0.0060 | 1.74E-09 | 450.9 |
| rs62331150 | 4 | 106069013 | T | G | 0.2333 | 0.0445 | 0.0071 | 4.09E-10 | 492.5 |
| rs77528541 | 4 | 126843504 | T | G | 0.1323 | -0.0549 | 0.0093 | 3.34E-09 | 481.4 |
| rs7697216 | 4 | 175828036 | C | T | 0.8916 | 0.1026 | 0.0094 | 1.55E-27 | 1416.9 |
| rs7736 | 5 | 314935 | C | T | 0.0569 | 0.0740 | 0.0130 | 1.33E-08 | 408.8 |
| rs6554679 | 5 | 1236690 | C | T | 0.7706 | 0.0422 | 0.0072 | 5.84E-09 | 437.3 |
| rs10054203 | 5 | 1279964 | C | G | 0.4196 | 0.0393 | 0.0062 | 2.71E-10 | 523.3 |
| rs2853669 | 5 | 1295349 | G | A | 0.2901 | -0.0673 | 0.0067 | 4.60E-24 | 1298.7 |
| rs12652713 | 5 | 16228197 | G | A | 0.5521 | -0.0421 | 0.0060 | 2.74E-12 | 610.1 |
| rs12519859 | 5 | 32581186 | A | G | 0.4713 | 0.0332 | 0.0061 | 4.31E-08 | 380.8 |
| rs11749176 | 5 | 44145931 | A | T | 0.1372 | -0.0532 | 0.0087 | 8.59E-10 | 466.0 |
| rs10941679 | 5 | 44706498 | G | A | 0.2769 | 0.1329 | 0.0068 | 3.82E-84 | 4944.5 |
| rs191615125 | 5 | 45375258 | C | T | 0.1438 | -0.0769 | 0.0089 | 6.18E-18 | 1013.8 |
| rs10941712 | 5 | 45917605 | T | C | 0.2450 | -0.0529 | 0.0071 | 1.02E-13 | 719.0 |
| rs139331653 | 5 | 45939294 | A | G | 0.0360 | 0.1133 | 0.0184 | 6.94E-10 | 619.8 |
| rs62390069 | 5 | 46400935 | G | A | 0.0375 | 0.0998 | 0.0168 | 2.60E-09 | 499.5 |
| rs72749841 | 5 | 49641645 | C | T | 0.1219 | -0.0648 | 0.0109 | 2.71E-09 | 624.4 |
| rs27279 | 5 | 50238519 | T | C | 0.4752 | -0.0429 | 0.0065 | 5.61E-11 | 637.6 |
| rs141930488 | 5 | 51248274 | A | G | 0.0236 | 0.1332 | 0.0241 | 3.46E-08 | 568.7 |
| rs7378815 | 5 | 55657706 | T | C | 0.1195 | -0.0560 | 0.0094 | 2.44E-09 | 459.3 |
| rs889310 | 5 | 55965167 | T | C | 0.5669 | 0.0394 | 0.0063 | 3.32E-10 | 531.1 |
| rs12653202 | 5 | 56016918 | C | A | 0.1817 | 0.1739 | 0.0080 | 9.85E-105 | 6304.0 |
| rs2408652 | 5 | 56271185 | A | G | 0.7889 | -0.0565 | 0.0073 | 1.43E-14 | 740.1 |
| rs17732378 | 5 | 56293009 | A | G | 0.5875 | 0.0370 | 0.0061 | 1.45E-09 | 460.4 |
| rs113778879 | 5 | 58241712 | T | C | 0.5684 | -0.0406 | 0.0064 | 1.70E-10 | 561.6 |
| rs4081859 | 5 | 81466669 | A | G | 0.7677 | 0.0530 | 0.0070 | 4.15E-14 | 695.4 |
| rs332529 | 5 | 90789470 | A | G | 0.1598 | -0.0641 | 0.0085 | 6.63E-14 | 766.7 |
| rs6860806 | 5 | 131640536 | G | A | 0.5438 | 0.0337 | 0.0061 | 2.66E-08 | 390.7 |
| rs6596100 | 5 | 132407058 | T | C | 0.2355 | -0.0411 | 0.0073 | 1.70E-08 | 423.1 |
| rs11135046 | 5 | 158230013 | T | G | 0.5390 | -0.0695 | 0.0060 | 4.23E-31 | 1673.7 |
| rs10074269 | 5 | 169591460 | C | T | 0.3361 | 0.0392 | 0.0064 | 1.09E-09 | 477.1 |
| rs418053 | 6 | 13713366 | C | G | 0.5558 | -0.0483 | 0.0061 | 1.56E-15 | 802.0 |
| rs3819405 | 6 | 16399557 | T | C | 0.3678 | -0.0424 | 0.0067 | 2.38E-10 | 581.2 |
| rs2223621 | 6 | 20621238 | C | T | 0.6095 | -0.0375 | 0.0063 | 2.01E-09 | 465.5 |
| rs7760611 | 6 | 21903533 | C | T | 0.5435 | 0.0363 | 0.0060 | 1.52E-09 | 453.8 |
| rs71557345 | 6 | 26680698 | A | G | 0.0778 | -0.0720 | 0.0126 | 1.14E-08 | 517.1 |
| rs1836962 | 6 | 81105862 | G | T | 0.5509 | 0.0393 | 0.0061 | 9.80E-11 | 530.1 |
| rs1361549 | 6 | 82287685 | A | G | 0.4116 | -0.0497 | 0.0061 | 3.12E-16 | 830.7 |
| rs4339481 | 6 | 130382653 | T | A | 0.6831 | 0.0452 | 0.0064 | 1.53E-12 | 614.0 |
| rs2121348 | 6 | 149595505 | C | T | 0.1949 | -0.0415 | 0.0075 | 3.66E-08 | 375.0 |
| rs60954078 | 6 | 151955914 | G | A | 0.0789 | 0.1781 | 0.0113 | 8.74E-56 | 3218.2 |
| rs6904031 | 6 | 152055978 | T | A | 0.0744 | 0.1351 | 0.0121 | 4.38E-29 | 1751.0 |
| rs2813549 | 6 | 152441239 | T | C | 0.7757 | 0.0589 | 0.0071 | 8.01E-17 | 840.6 |
| rs7971 | 7 | 21940960 | G | A | 0.3475 | -0.0355 | 0.0063 | 1.69E-08 | 396.9 |
| rs2075881 | 7 | 91734552 | C | T | 0.3984 | 0.0404 | 0.0061 | 4.72E-11 | 542.7 |
| rs2188648 | 7 | 94111956 | C | T | 0.2883 | 0.0486 | 0.0066 | 2.21E-13 | 674.7 |
| rs1541409 | 7 | 98027580 | T | C | 0.2316 | -0.0403 | 0.0071 | 1.59E-08 | 401.6 |
| rs71559437 | 7 | 101552440 | A | G | 0.1094 | -0.0578 | 0.0095 | 1.19E-09 | 452.5 |
| rs12706954 | 7 | 130656911 | T | C | 0.3653 | -0.0432 | 0.0064 | 1.44E-11 | 600.5 |
| rs68056147 | 7 | 130674481 | A | G | 0.3170 | 0.0528 | 0.0067 | 2.97E-15 | 838.1 |
| rs11977670 | 7 | 139942304 | A | G | 0.4421 | 0.0531 | 0.0061 | 2.69E-18 | 966.1 |
| rs62485509 | 7 | 144048902 | T | G | 0.2200 | -0.0517 | 0.0076 | 9.94E-12 | 638.3 |
| rs13256025 | 8 | 25831778 | T | C | 0.1965 | 0.0446 | 0.0079 | 1.41E-08 | 436.6 |
| rs7463114 | 8 | 29507094 | C | T | 0.6609 | -0.0597 | 0.0064 | 6.16E-21 | 1111.4 |
| rs4286946 | 8 | 36849946 | G | C | 0.1530 | -0.0792 | 0.0080 | 7.41E-23 | 1130.1 |
| rs1511243 | 8 | 76230943 | G | A | 0.8359 | 0.0778 | 0.0080 | 2.20E-22 | 1155.9 |
| rs72658071 | 8 | 76305785 | T | A | 0.0955 | 0.1174 | 0.0103 | 7.16E-30 | 1658.9 |
| rs1533366 | 8 | 76378165 | T | G | 0.3459 | -0.0435 | 0.0063 | 4.33E-12 | 595.4 |
| rs62517052 | 8 | 102483100 | C | T | 0.1044 | 0.0666 | 0.0102 | 7.56E-11 | 577.6 |
| rs12546444 | 8 | 106358620 | T | A | 0.0952 | -0.0718 | 0.0106 | 1.08E-11 | 617.3 |
| rs13277568 | 8 | 116679547 | G | A | 0.3643 | -0.0356 | 0.0064 | 2.23E-08 | 407.7 |
| rs13267382 | 8 | 117209548 | G | A | 0.6388 | -0.0430 | 0.0063 | 7.65E-12 | 592.0 |
| rs58847541 | 8 | 124610166 | A | G | 0.1546 | 0.0617 | 0.0084 | 1.91E-13 | 692.9 |
| rs970822 | 8 | 124738742 | T | C | 0.4122 | 0.0429 | 0.0061 | 2.88E-12 | 619.5 |
| rs10096351 | 8 | 128372172 | G | A | 0.5689 | 0.1059 | 0.0060 | 3.77E-69 | 3845.0 |
| rs1016578 | 8 | 129199566 | A | G | 0.1716 | 0.0632 | 0.0079 | 1.21E-15 | 789.6 |
| rs3217992 | 9 | 22003223 | T | C | 0.3569 | -0.0535 | 0.0062 | 6.53E-18 | 913.3 |
| rs4742903 | 9 | 106856793 | C | G | 0.5637 | 0.0336 | 0.0060 | 2.58E-08 | 385.1 |
| rs55760189 | 9 | 110298930 | G | A | 0.1429 | 0.0530 | 0.0091 | 6.00E-09 | 478.5 |
| rs10978911 | 9 | 110306944 | C | G | 0.1451 | 0.0790 | 0.0088 | 2.21E-19 | 1076.8 |
| rs10816625 | 9 | 110837073 | G | A | 0.0686 | 0.1122 | 0.0122 | 4.20E-20 | 1120.1 |
| rs1999456 | 9 | 110877304 | G | C | 0.5513 | 0.0454 | 0.0060 | 4.35E-14 | 707.7 |
| rs630965 | 9 | 110885479 | T | C | 0.6427 | 0.1007 | 0.0062 | 1.95E-59 | 3251.2 |
| rs1895062 | 9 | 119313486 | G | A | 0.3913 | -0.0430 | 0.0061 | 2.51E-12 | 612.8 |
| rs10760444 | 9 | 129396434 | A | G | 0.5558 | -0.0345 | 0.0060 | 9.63E-09 | 409.6 |
| rs67801543 | 10 | 9108324 | T | C | 0.1253 | 0.0575 | 0.0091 | 3.11E-10 | 503.8 |
| rs2009607 | 10 | 21978590 | T | A | 0.0578 | 0.1024 | 0.0166 | 7.45E-10 | 794.8 |
| rs7072776 | 10 | 22032942 | G | A | 0.7021 | -0.0628 | 0.0066 | 2.51E-21 | 1146.5 |
| rs55716112 | 10 | 22521736 | G | T | 0.0295 | 0.1423 | 0.0194 | 2.15E-13 | 807.5 |
| rs12260388 | 10 | 22863425 | G | A | 0.9668 | 0.0951 | 0.0161 | 3.70E-09 | 403.2 |
| rs10995201 | 10 | 64299890 | G | A | 0.1419 | -0.1251 | 0.0085 | 1.60E-49 | 2656.6 |
| rs12765365 | 10 | 64848937 | C | T | 0.0341 | -0.0973 | 0.0169 | 9.05E-09 | 433.0 |
| rs719338 | 10 | 80851257 | T | G | 0.5972 | -0.0780 | 0.0061 | 2.81E-37 | 2039.8 |
| rs10762849 | 10 | 80886061 | T | C | 0.1725 | 0.0780 | 0.0081 | 3.52E-22 | 1209.9 |
| rs10885405 | 10 | 114777670 | T | C | 0.4660 | 0.0454 | 0.0060 | 3.30E-14 | 713.1 |
| rs1467576 | 10 | 114888728 | C | G | 0.6268 | 0.0337 | 0.0062 | 4.59E-08 | 370.0 |
| rs12250948 | 10 | 115128491 | C | T | 0.7731 | -0.0539 | 0.0072 | 7.78E-14 | 707.6 |
| rs2420941 | 10 | 123229626 | G | T | 0.4850 | -0.0382 | 0.0061 | 2.95E-10 | 507.6 |
| rs72832370 | 10 | 123294692 | T | C | 0.0726 | -0.0879 | 0.0116 | 3.47E-14 | 723.5 |
| rs4752569 | 10 | 123331690 | T | A | 0.5318 | 0.1913 | 0.0062 | 1.00E-200 | 12897.1 |
| rs61390772 | 10 | 123368849 | T | C | 0.0365 | 0.1442 | 0.0178 | 4.66E-16 | 1019.0 |
| rs72832402 | 10 | 123395154 | C | G | 0.0829 | -0.0854 | 0.0106 | 6.24E-16 | 771.7 |
| rs61874140 | 10 | 123435982 | G | A | 0.0266 | 0.1455 | 0.0196 | 1.20E-13 | 762.5 |
| rs1909666 | 10 | 123444567 | G | A | 0.9115 | -0.0685 | 0.0106 | 1.00E-10 | 526.4 |
| rs12241931 | 10 | 123493274 | T | C | 0.0902 | 0.0868 | 0.0107 | 4.25E-16 | 860.7 |
| rs6597981 | 11 | 803017 | G | A | 0.5306 | 0.0452 | 0.0060 | 4.65E-14 | 707.5 |
| rs1973765 | 11 | 1898664 | C | T | 0.3833 | -0.0766 | 0.0062 | 1.77E-35 | 1933.0 |
| rs10838267 | 11 | 44368892 | A | G | 0.5479 | 0.0330 | 0.0060 | 4.51E-08 | 375.5 |
| rs3892696 | 11 | 65582341 | C | G | 0.4582 | -0.0442 | 0.0060 | 1.59E-13 | 674.2 |
| rs148893083 | 11 | 69072697 | T | C | 0.0199 | 0.1561 | 0.0224 | 3.20E-12 | 660.2 |
| rs506516 | 11 | 69306595 | G | T | 0.6707 | 0.0574 | 0.0064 | 2.24E-19 | 1013.8 |
| rs78540526 | 11 | 69331418 | T | C | 0.0987 | 0.2813 | 0.0109 | 2.09E-147 | 9921.5 |
| rs7939702 | 11 | 129243417 | G | T | 0.8506 | -0.0487 | 0.0087 | 1.87E-08 | 418.2 |
| rs11822830 | 11 | 129461016 | G | A | 0.6014 | 0.0465 | 0.0061 | 2.72E-14 | 720.3 |
| rs12422552 | 12 | 14413931 | C | G | 0.2670 | 0.0584 | 0.0068 | 8.12E-18 | 927.6 |
| rs7297051 | 12 | 28174817 | T | C | 0.2174 | -0.1234 | 0.0071 | 2.38E-67 | 3619.1 |
| rs11049420 | 12 | 28346112 | A | G | 0.3030 | -0.0574 | 0.0065 | 8.46E-19 | 968.1 |
| rs11116495 | 12 | 85005151 | G | A | 0.4981 | 0.0342 | 0.0060 | 1.29E-08 | 406.8 |
| rs17356907 | 12 | 96027759 | G | A | 0.2812 | -0.0881 | 0.0066 | 8.11E-41 | 2187.5 |
| rs4378452 | 12 | 111504033 | C | T | 0.3502 | -0.0348 | 0.0062 | 2.36E-08 | 382.9 |
| rs3741698 | 12 | 115109223 | G | C | 0.2832 | 0.0405 | 0.0067 | 1.72E-09 | 462.1 |
| rs2454399 | 12 | 115835836 | C | T | 0.3953 | -0.0833 | 0.0061 | 1.13E-42 | 2309.5 |
| rs11571833 | 13 | 32972626 | T | A | 0.0105 | 0.2764 | 0.0327 | 2.63E-17 | 1108.3 |
| rs17181761 | 13 | 73811471 | C | A | 0.3292 | 0.0426 | 0.0064 | 3.00E-11 | 557.6 |
| rs4885096 | 13 | 73967507 | C | T | 0.7056 | 0.0375 | 0.0065 | 7.38E-09 | 406.5 |
| rs7149262 | 14 | 37136545 | A | C | 0.1955 | -0.0725 | 0.0074 | 1.27E-22 | 1149.2 |
| rs2253012 | 14 | 37228504 | T | C | 0.4213 | 0.0388 | 0.0062 | 5.04E-10 | 510.2 |
| rs12894297 | 14 | 68650548 | A | C | 0.8257 | -0.0606 | 0.0080 | 3.13E-14 | 735.1 |
| rs11624333 | 14 | 68979835 | C | T | 0.2442 | -0.0956 | 0.0069 | 1.55E-43 | 2349.1 |
| rs74189900 | 14 | 91759767 | A | G | 0.7230 | 0.0493 | 0.0081 | 1.03E-09 | 675.9 |
| rs941764 | 14 | 91841069 | G | A | 0.3534 | 0.0492 | 0.0063 | 4.11E-15 | 770.3 |
| rs78440108 | 14 | 93070286 | T | C | 0.1643 | -0.0614 | 0.0081 | 3.89E-14 | 720.9 |
| rs4983544 | 14 | 105213978 | G | T | 0.4724 | 0.0367 | 0.0061 | 2.04E-09 | 467.2 |
| rs12594752 | 15 | 91531995 | T | C | 0.1285 | -0.0766 | 0.0089 | 6.74E-18 | 914.3 |
| rs2601774 | 16 | 4027749 | G | A | 0.8385 | 0.0503 | 0.0084 | 1.90E-09 | 476.6 |
| rs17271951 | 16 | 52538040 | C | T | 0.2939 | 0.2080 | 0.0067 | 1.00E-200 | 12702.6 |
| rs150540840 | 16 | 52594838 | T | G | 0.0466 | -0.0945 | 0.0147 | 1.37E-10 | 551.8 |
| rs62048402 | 16 | 53803223 | A | G | 0.3981 | -0.0594 | 0.0061 | 1.94E-22 | 1175.8 |
| rs7184573 | 16 | 53861592 | A | G | 0.3424 | -0.0478 | 0.0063 | 3.20E-14 | 716.0 |
| rs28539243 | 16 | 54682064 | A | G | 0.5029 | 0.0465 | 0.0061 | 1.52E-14 | 753.2 |
| rs7499149 | 16 | 80648327 | G | A | 0.2448 | 0.0809 | 0.0070 | 1.42E-30 | 1683.8 |
| rs4843504 | 16 | 87032855 | C | T | 0.7443 | 0.0426 | 0.0069 | 6.26E-10 | 480.9 |
| rs79461387 | 17 | 29168077 | T | G | 0.2528 | -0.0418 | 0.0069 | 1.22E-09 | 459.7 |
| rs62064364 | 17 | 43654468 | T | C | 0.1926 | -0.0486 | 0.0076 | 1.64E-10 | 511.5 |
| rs78381082 | 17 | 44182983 | G | A | 0.1559 | -0.0543 | 0.0085 | 2.11E-10 | 539.0 |
| rs2787486 | 17 | 53209774 | C | A | 0.2838 | -0.0715 | 0.0066 | 1.46E-27 | 1446.6 |
| rs11652463 | 17 | 70405095 | G | C | 0.3065 | -0.0395 | 0.0072 | 4.19E-08 | 461.6 |
| rs8082452 | 17 | 77771548 | G | T | 0.4443 | -0.0418 | 0.0062 | 2.28E-11 | 598.9 |
| rs12962334 | 18 | 20477934 | C | G | 0.6715 | -0.0377 | 0.0064 | 3.83E-09 | 435.4 |
| rs7240205 | 18 | 24156018 | T | C | 0.6026 | -0.0373 | 0.0062 | 2.34E-09 | 462.5 |
| rs527616 | 18 | 24337424 | G | C | 0.6306 | 0.0512 | 0.0062 | 1.68E-16 | 848.4 |
| rs170801 | 18 | 24500899 | A | C | 0.2823 | -0.0635 | 0.0068 | 7.28E-21 | 1136.7 |
| rs72931898 | 18 | 29981526 | A | G | 0.0446 | -0.1035 | 0.0145 | 9.68E-13 | 634.3 |
| rs9954058 | 18 | 42411803 | C | G | 0.0668 | -0.0837 | 0.0118 | 1.30E-12 | 606.9 |
| rs9952980 | 18 | 42888797 | C | T | 0.3379 | -0.0485 | 0.0063 | 1.08E-14 | 732.6 |
| rs78269692 | 19 | 13158277 | C | T | 0.0444 | 0.0882 | 0.0145 | 1.14E-09 | 459.0 |
| rs1531212 | 19 | 13951830 | A | G | 0.2271 | -0.0399 | 0.0072 | 2.65E-08 | 387.7 |
| rs56069439 | 19 | 17393925 | A | C | 0.3049 | 0.0376 | 0.0065 | 8.98E-09 | 415.9 |
| rs8105994 | 19 | 18593553 | C | T | 0.3339 | -0.0709 | 0.0063 | 4.77E-29 | 1554.8 |
| rs4808961 | 19 | 19577215 | G | C | 0.3751 | 0.0405 | 0.0062 | 8.48E-11 | 535.7 |
| rs56681946 | 19 | 44283031 | C | T | 0.3632 | 0.0621 | 0.0064 | 2.34E-22 | 1242.2 |
| rs61373376 | 19 | 46183586 | T | C | 0.1689 | 0.0523 | 0.0085 | 6.30E-10 | 534.0 |
| rs16991615 | 20 | 5948227 | A | G | 0.0646 | 0.0753 | 0.0122 | 7.48E-10 | 476.3 |
| rs6122906 | 20 | 48945911 | G | A | 0.1903 | 0.0469 | 0.0077 | 1.34E-09 | 471.9 |
| rs13039563 | 20 | 52296849 | A | G | 0.2452 | 0.0424 | 0.0072 | 3.05E-09 | 462.5 |
| rs2822999 | 21 | 16364756 | G | T | 0.1769 | 0.0583 | 0.0083 | 2.86E-12 | 687.4 |
| rs2403907 | 21 | 16574455 | A | C | 0.2990 | -0.0784 | 0.0065 | 1.38E-33 | 1796.5 |
| rs9808759 | 21 | 47780223 | C | T | 0.9196 | -0.0665 | 0.0114 | 5.84E-09 | 455.1 |
| rs62237573 | 22 | 28552698 | T | C | 0.0126 | 0.4281 | 0.0318 | 2.00E-41 | 3176.0 |
| rs9620778 | 22 | 28620584 | A | G | 0.1036 | 0.0658 | 0.0103 | 1.47E-10 | 559.3 |
| rs75174485 | 22 | 29078705 | G | T | 0.0205 | 0.2988 | 0.0270 | 1.58E-28 | 2498.9 |
| rs5997390 | 22 | 29135543 | A | G | 0.1001 | 0.0731 | 0.0104 | 2.42E-12 | 670.2 |
| rs132365 | 22 | 29599486 | C | G | 0.9793 | -0.2400 | 0.0251 | 1.08E-21 | 1628.5 |
| rs140091 | 22 | 30110546 | A | G | 0.0157 | 0.1545 | 0.0263 | 4.16E-09 | 514.5 |
| rs738321 | 22 | 38568833 | G | C | 0.3633 | -0.0475 | 0.0062 | 1.57E-14 | 725.8 |
| rs12628403 | 22 | 39358037 | C | A | 0.1028 | 0.0806 | 0.0117 | 6.40E-12 | 833.9 |
| rs9611271 | 22 | 40526214 | G | A | 0.3409 | 0.0364 | 0.0064 | 1.67E-08 | 413.3 |
| rs5995875 | 22 | 40960692 | T | C | 0.1192 | 0.1167 | 0.0095 | 9.57E-35 | 1991.6 |
| rs73161324 | 22 | 42038786 | T | C | 0.0644 | 0.0817 | 0.0129 | 2.47E-10 | 558.7 |
| rs28512361 | 22 | 46283297 | A | G | 0.1188 | 0.0612 | 0.0104 | 3.52E-09 | 544.5 |

F-statistic was calculated using the following formulas: F=R^2 (n-2)⁄(1-R^2) and R^2=2×MAF×(1-MAF)×β^2, where F represents F-statistic, R^2 represents the phenotypic variance explained by a genetic instrument, N is the sample size, β is the estimated genetic association of SNP with the exposure, MAF is the minor allele frequency. SNP: single nucleotide polymorphisms.

**Table S4. Characteristics of GWAS-identified Malignant neoplasm of thyroid gland-associated genetic instruments.**

| **SNP** | **Chromosome** | **Position** | **Effect allele** | **Other allele** | **Effect allele frequency** | **Beta** | **Standard Error** | ***P*-value** | ***F*-statistic** |
| --- | --- | --- | --- | --- | --- | --- | --- | --- | --- |
| rs722082 | 2 | 217374516 | G | A | 0.0794 | 0.3310 | 0.0564 | 4.50E-09 | 11301.4 |
| rs2373058 | 2 | 217385826 | C | G | 0.2331 | 0.2342 | 0.0381 | 8.17E-10 | 13897.9 |
| rs10054203 | 5 | 1279849 | C | G | 0.4593 | 0.1854 | 0.0334 | 2.85E-08 | 12062.7 |
| rs4129579 | 8 | 32440335 | A | G | 0.3570 | 0.1974 | 0.0343 | 8.31E-09 | 12647.5 |
| rs10817378 | 9 | 97292147 | T | C | 0.2233 | 0.2179 | 0.0387 | 1.80E-08 | 11627.9 |
| rs10982766 | 9 | 97566280 | T | G | 0.4906 | -0.1891 | 0.0335 | 1.59E-08 | 12638.3 |
| rs7034310 | 9 | 97764875 | G | A | 0.3233 | -0.2101 | 0.0367 | 1.01E-08 | 13674.0 |
| rs7853349 | 9 | 97887977 | T | A | 0.4402 | -0.2041 | 0.0340 | 1.92E-09 | 14555.2 |
| rs2755193 | 14 | 36049416 | T | G | 0.4335 | -0.1858 | 0.0340 | 4.51E-08 | 11986.8 |
| rs17293443 | 15 | 67145525 | C | T | 0.2707 | 0.2519 | 0.0365 | 5.42E-12 | 17855.6 |
| rs62237573 | 22 | 28156710 | T | C | 0.0165 | 0.6760 | 0.1078 | 3.65E-10 | 10486.7 |

F-statistic was calculated using the following formulas: F=R^2 (n-2)⁄(1-R^2) and R^2=2×MAF×(1-MAF)×β^2, where F represents F-statistic, R^2 represents the phenotypic variance explained by a genetic instrument, N is the sample size, β is the estimated genetic association of SNP with the exposure, MAF is the minor allele frequency. SNP: single nucleotide polymorphisms.

**Table S5. Characteristics of GWAS-identified Benign neoplasm of thyroid gland-associated genetic instruments.**

| **SNP** | **Chromosome** | **Position** | **Effect allele** | **Other allele** | **Effect allele frequency** | **Beta** | **Standard Error** | ***P*-value** | ***F*-statistic** |
| --- | --- | --- | --- | --- | --- | --- | --- | --- | --- |
| rs13259143 | 8 | 8964127 | G | A | 0.4503 | 0.2211 | 0.0474 | 3.11E-06 | 17226.6 |
| rs10983655 | 9 | 97762040 | C | T | 0.5232 | -0.2612 | 0.0472 | 3.11E-08 | 24471.0 |
| rs12266386 | 10 | 95138039 | C | T | 0.4277 | 0.2250 | 0.0473 | 1.94E-06 | 17646.4 |
| rs4815130 | 20 | 22655214 | A | G | 0.7468 | -0.2539 | 0.0524 | 1.29E-06 | 17357.3 |

F-statistic was calculated using the following formulas: F=R^2 (n-2)⁄(1-R^2) and R^2=2×MAF×(1-MAF)×β^2, where F represents F-statistic, R^2 represents the phenotypic variance explained by a genetic instrument, N is the sample size, β is the estimated genetic association of SNP with the exposure, MAF is the minor allele frequency. SNP: single nucleotide polymorphisms.

**Table S6. The result of leave-one-out approach for Overall Breast Cancer and Malignant neoplasm of thyroid gland**

| **Exposure** | **Outcome** | **SNP** | **Beta** | **Standard error** | **OR (95%CI)** | **P-value** |
| --- | --- | --- | --- | --- | --- | --- |
| Overall Breast Cancer | Malignant neoplasm of thyroid gland | rs10022462 | 0.26 | 0.06 | 1.293 (1.145-1.461) | 3.65E-05 |
| Overall Breast Cancer | Malignant neoplasm of thyroid gland | rs10074269 | 0.25 | 0.06 | 1.287 (1.140-1.454) | 4.81E-05 |
| Overall Breast Cancer | Malignant neoplasm of thyroid gland | rs10096351 | 0.25 | 0.06 | 1.285 (1.136-1.453) | 6.59E-05 |
| Overall Breast Cancer | Malignant neoplasm of thyroid gland | rs1016578 | 0.26 | 0.06 | 1.293 (1.144-1.461) | 3.79E-05 |
| Overall Breast Cancer | Malignant neoplasm of thyroid gland | rs10760444 | 0.26 | 0.06 | 1.293 (1.145-1.461) | 3.63E-05 |
| Overall Breast Cancer | Malignant neoplasm of thyroid gland | rs10762849 | 0.26 | 0.06 | 1.295 (1.146-1.464) | 3.33E-05 |
| Overall Breast Cancer | Malignant neoplasm of thyroid gland | rs10816625 | 0.26 | 0.06 | 1.291 (1.142-1.459) | 4.27E-05 |
| Overall Breast Cancer | Malignant neoplasm of thyroid gland | rs10838267 | 0.26 | 0.06 | 1.294 (1.145-1.462) | 3.49E-05 |
| Overall Breast Cancer | Malignant neoplasm of thyroid gland | rs10885405 | 0.25 | 0.06 | 1.290 (1.142-1.458) | 4.44E-05 |
| Overall Breast Cancer | Malignant neoplasm of thyroid gland | rs10941679 | 0.27 | 0.06 | 1.304 (1.153-1.475) | 2.36E-05 |
| Overall Breast Cancer | Malignant neoplasm of thyroid gland | rs10941712 | 0.26 | 0.06 | 1.299 (1.150-1.467) | 2.46E-05 |
| Overall Breast Cancer | Malignant neoplasm of thyroid gland | rs10978911 | 0.26 | 0.06 | 1.295 (1.146-1.463) | 3.41E-05 |
| Overall Breast Cancer | Malignant neoplasm of thyroid gland | rs11049420 | 0.26 | 0.06 | 1.292 (1.143-1.460) | 4.14E-05 |
| Overall Breast Cancer | Malignant neoplasm of thyroid gland | rs11116495 | 0.25 | 0.06 | 1.290 (1.142-1.458) | 4.29E-05 |
| Overall Breast Cancer | Malignant neoplasm of thyroid gland | rs11117758 | 0.26 | 0.06 | 1.291 (1.143-1.459) | 4.15E-05 |
| Overall Breast Cancer | Malignant neoplasm of thyroid gland | rs11205303 | 0.26 | 0.06 | 1.296 (1.147-1.464) | 3.14E-05 |
| Overall Breast Cancer | Malignant neoplasm of thyroid gland | rs11249433 | 0.26 | 0.06 | 1.299 (1.149-1.469) | 2.99E-05 |
| Overall Breast Cancer | Malignant neoplasm of thyroid gland | rs11264454 | 0.25 | 0.06 | 1.285 (1.138-1.451) | 5.04E-05 |
| Overall Breast Cancer | Malignant neoplasm of thyroid gland | rs113577745 | 0.26 | 0.06 | 1.293 (1.145-1.461) | 3.54E-05 |
| Overall Breast Cancer | Malignant neoplasm of thyroid gland | rs11571833 | 0.26 | 0.06 | 1.294 (1.145-1.462) | 3.61E-05 |
| Overall Breast Cancer | Malignant neoplasm of thyroid gland | rs11583393 | 0.26 | 0.06 | 1.292 (1.144-1.460) | 3.93E-05 |
| Overall Breast Cancer | Malignant neoplasm of thyroid gland | rs11624333 | 0.27 | 0.06 | 1.304 (1.154-1.473) | 2.05E-05 |
| Overall Breast Cancer | Malignant neoplasm of thyroid gland | rs11652463 | 0.26 | 0.06 | 1.294 (1.146-1.462) | 3.37E-05 |
| Overall Breast Cancer | Malignant neoplasm of thyroid gland | rs11680449 | 0.26 | 0.06 | 1.292 (1.143-1.460) | 4.01E-05 |
| Overall Breast Cancer | Malignant neoplasm of thyroid gland | rs11684853 | 0.26 | 0.06 | 1.295 (1.146-1.463) | 3.41E-05 |
| Overall Breast Cancer | Malignant neoplasm of thyroid gland | rs11693806 | 0.22 | 0.06 | 1.243 (1.115-1.384) | 8.30E-05 |
| Overall Breast Cancer | Malignant neoplasm of thyroid gland | rs11749176 | 0.26 | 0.06 | 1.292 (1.144-1.460) | 3.89E-05 |
| Overall Breast Cancer | Malignant neoplasm of thyroid gland | rs11822830 | 0.25 | 0.06 | 1.290 (1.142-1.458) | 4.43E-05 |
| Overall Breast Cancer | Malignant neoplasm of thyroid gland | rs11903787 | 0.25 | 0.06 | 1.290 (1.142-1.457) | 4.40E-05 |
| Overall Breast Cancer | Malignant neoplasm of thyroid gland | rs11977670 | 0.26 | 0.06 | 1.298 (1.149-1.466) | 2.75E-05 |
| Overall Breast Cancer | Malignant neoplasm of thyroid gland | rs12091730 | 0.26 | 0.06 | 1.295 (1.146-1.463) | 3.29E-05 |
| Overall Breast Cancer | Malignant neoplasm of thyroid gland | rs12250948 | 0.26 | 0.06 | 1.292 (1.143-1.460) | 4.00E-05 |
| Overall Breast Cancer | Malignant neoplasm of thyroid gland | rs12260388 | 0.26 | 0.06 | 1.296 (1.148-1.464) | 2.86E-05 |
| Overall Breast Cancer | Malignant neoplasm of thyroid gland | rs12422552 | 0.25 | 0.06 | 1.288 (1.140-1.456) | 4.78E-05 |
| Overall Breast Cancer | Malignant neoplasm of thyroid gland | rs12479355 | 0.26 | 0.06 | 1.291 (1.143-1.459) | 4.11E-05 |
| Overall Breast Cancer | Malignant neoplasm of thyroid gland | rs12493607 | 0.26 | 0.06 | 1.295 (1.146-1.463) | 3.38E-05 |
| Overall Breast Cancer | Malignant neoplasm of thyroid gland | rs12519859 | 0.25 | 0.06 | 1.290 (1.141-1.457) | 4.42E-05 |
| Overall Breast Cancer | Malignant neoplasm of thyroid gland | rs12594752 | 0.25 | 0.06 | 1.286 (1.139-1.454) | 5.30E-05 |
| Overall Breast Cancer | Malignant neoplasm of thyroid gland | rs12628403 | 0.26 | 0.06 | 1.292 (1.144-1.461) | 3.90E-05 |
| Overall Breast Cancer | Malignant neoplasm of thyroid gland | rs12652713 | 0.26 | 0.06 | 1.295 (1.146-1.463) | 3.36E-05 |
| Overall Breast Cancer | Malignant neoplasm of thyroid gland | rs12653202 | 0.26 | 0.06 | 1.298 (1.147-1.468) | 3.56E-05 |
| Overall Breast Cancer | Malignant neoplasm of thyroid gland | rs12706954 | 0.25 | 0.06 | 1.290 (1.141-1.457) | 4.48E-05 |
| Overall Breast Cancer | Malignant neoplasm of thyroid gland | rs12765365 | 0.26 | 0.06 | 1.292 (1.144-1.460) | 3.80E-05 |
| Overall Breast Cancer | Malignant neoplasm of thyroid gland | rs12894297 | 0.25 | 0.06 | 1.289 (1.141-1.457) | 4.50E-05 |
| Overall Breast Cancer | Malignant neoplasm of thyroid gland | rs12962334 | 0.26 | 0.06 | 1.294 (1.145-1.462) | 3.45E-05 |
| Overall Breast Cancer | Malignant neoplasm of thyroid gland | rs13039563 | 0.26 | 0.06 | 1.297 (1.149-1.464) | 2.71E-05 |
| Overall Breast Cancer | Malignant neoplasm of thyroid gland | rs13066793 | 0.26 | 0.06 | 1.294 (1.145-1.462) | 3.53E-05 |
| Overall Breast Cancer | Malignant neoplasm of thyroid gland | rs132365 | 0.23 | 0.06 | 1.258 (1.119-1.414) | 1.22E-04 |
| Overall Breast Cancer | Malignant neoplasm of thyroid gland | rs13256025 | 0.26 | 0.06 | 1.291 (1.143-1.459) | 4.15E-05 |
| Overall Breast Cancer | Malignant neoplasm of thyroid gland | rs13267382 | 0.25 | 0.06 | 1.290 (1.142-1.458) | 4.43E-05 |
| Overall Breast Cancer | Malignant neoplasm of thyroid gland | rs13277568 | 0.26 | 0.06 | 1.296 (1.147-1.464) | 2.97E-05 |
| Overall Breast Cancer | Malignant neoplasm of thyroid gland | rs1361549 | 0.26 | 0.06 | 1.298 (1.149-1.466) | 2.76E-05 |
| Overall Breast Cancer | Malignant neoplasm of thyroid gland | rs1375631 | 0.25 | 0.06 | 1.290 (1.142-1.458) | 4.30E-05 |
| Overall Breast Cancer | Malignant neoplasm of thyroid gland | rs139331653 | 0.26 | 0.06 | 1.293 (1.144-1.461) | 3.74E-05 |
| Overall Breast Cancer | Malignant neoplasm of thyroid gland | rs140091 | 0.24 | 0.06 | 1.276 (1.131-1.439) | 7.54E-05 |
| Overall Breast Cancer | Malignant neoplasm of thyroid gland | rs141930488 | 0.26 | 0.06 | 1.293 (1.144-1.460) | 3.73E-05 |
| Overall Breast Cancer | Malignant neoplasm of thyroid gland | rs1467576 | 0.26 | 0.06 | 1.293 (1.145-1.461) | 3.66E-05 |
| Overall Breast Cancer | Malignant neoplasm of thyroid gland | rs148893083 | 0.25 | 0.06 | 1.289 (1.141-1.456) | 4.60E-05 |
| Overall Breast Cancer | Malignant neoplasm of thyroid gland | rs150540840 | 0.26 | 0.06 | 1.299 (1.150-1.467) | 2.63E-05 |
| Overall Breast Cancer | Malignant neoplasm of thyroid gland | rs1511243 | 0.26 | 0.06 | 1.297 (1.147-1.465) | 3.12E-05 |
| Overall Breast Cancer | Malignant neoplasm of thyroid gland | rs1531212 | 0.25 | 0.06 | 1.289 (1.141-1.456) | 4.56E-05 |
| Overall Breast Cancer | Malignant neoplasm of thyroid gland | rs1533366 | 0.26 | 0.06 | 1.292 (1.143-1.460) | 3.94E-05 |
| Overall Breast Cancer | Malignant neoplasm of thyroid gland | rs1541409 | 0.26 | 0.06 | 1.291 (1.142-1.459) | 4.20E-05 |
| Overall Breast Cancer | Malignant neoplasm of thyroid gland | rs16991615 | 0.25 | 0.06 | 1.288 (1.141-1.455) | 4.41E-05 |
| Overall Breast Cancer | Malignant neoplasm of thyroid gland | rs1707302 | 0.26 | 0.06 | 1.295 (1.146-1.463) | 3.21E-05 |
| Overall Breast Cancer | Malignant neoplasm of thyroid gland | rs170801 | 0.26 | 0.06 | 1.303 (1.154-1.471) | 1.85E-05 |
| Overall Breast Cancer | Malignant neoplasm of thyroid gland | rs17181761 | 0.26 | 0.06 | 1.295 (1.146-1.463) | 3.30E-05 |
| Overall Breast Cancer | Malignant neoplasm of thyroid gland | rs17271951 | 0.25 | 0.06 | 1.284 (1.132-1.456) | 1.01E-04 |
| Overall Breast Cancer | Malignant neoplasm of thyroid gland | rs17356907 | 0.26 | 0.06 | 1.297 (1.147-1.466) | 3.30E-05 |
| Overall Breast Cancer | Malignant neoplasm of thyroid gland | rs17732378 | 0.25 | 0.06 | 1.289 (1.141-1.457) | 4.54E-05 |
| Overall Breast Cancer | Malignant neoplasm of thyroid gland | rs1836962 | 0.25 | 0.06 | 1.287 (1.140-1.454) | 4.89E-05 |
| Overall Breast Cancer | Malignant neoplasm of thyroid gland | rs1895062 | 0.26 | 0.06 | 1.294 (1.145-1.462) | 3.50E-05 |
| Overall Breast Cancer | Malignant neoplasm of thyroid gland | rs1909666 | 0.25 | 0.06 | 1.287 (1.140-1.454) | 4.77E-05 |
| Overall Breast Cancer | Malignant neoplasm of thyroid gland | rs1973765 | 0.25 | 0.06 | 1.281 (1.134-1.448) | 6.98E-05 |
| Overall Breast Cancer | Malignant neoplasm of thyroid gland | rs2010610 | 0.26 | 0.06 | 1.294 (1.145-1.461) | 3.59E-05 |
| Overall Breast Cancer | Malignant neoplasm of thyroid gland | rs2016394 | 0.25 | 0.06 | 1.290 (1.142-1.458) | 4.32E-05 |
| Overall Breast Cancer | Malignant neoplasm of thyroid gland | rs2028195 | 0.26 | 0.06 | 1.295 (1.146-1.463) | 3.24E-05 |
| Overall Breast Cancer | Malignant neoplasm of thyroid gland | rs2075881 | 0.26 | 0.06 | 1.295 (1.146-1.463) | 3.23E-05 |
| Overall Breast Cancer | Malignant neoplasm of thyroid gland | rs2121348 | 0.26 | 0.06 | 1.294 (1.146-1.462) | 3.31E-05 |
| Overall Breast Cancer | Malignant neoplasm of thyroid gland | rs2141818 | 0.25 | 0.06 | 1.281 (1.135-1.446) | 6.25E-05 |
| Overall Breast Cancer | Malignant neoplasm of thyroid gland | rs2188648 | 0.26 | 0.06 | 1.295 (1.146-1.463) | 3.30E-05 |
| Overall Breast Cancer | Malignant neoplasm of thyroid gland | rs2223621 | 0.25 | 0.06 | 1.289 (1.141-1.456) | 4.60E-05 |
| Overall Breast Cancer | Malignant neoplasm of thyroid gland | rs2253012 | 0.26 | 0.06 | 1.292 (1.144-1.460) | 3.84E-05 |
| Overall Breast Cancer | Malignant neoplasm of thyroid gland | rs2403907 | 0.25 | 0.06 | 1.289 (1.141-1.457) | 4.85E-05 |
| Overall Breast Cancer | Malignant neoplasm of thyroid gland | rs2408652 | 0.26 | 0.06 | 1.295 (1.146-1.463) | 3.42E-05 |
| Overall Breast Cancer | Malignant neoplasm of thyroid gland | rs2420941 | 0.26 | 0.06 | 1.292 (1.143-1.460) | 4.02E-05 |
| Overall Breast Cancer | Malignant neoplasm of thyroid gland | rs2454399 | 0.27 | 0.06 | 1.309 (1.160-1.478) | 1.36E-05 |
| Overall Breast Cancer | Malignant neoplasm of thyroid gland | rs2601774 | 0.26 | 0.06 | 1.295 (1.146-1.463) | 3.22E-05 |
| Overall Breast Cancer | Malignant neoplasm of thyroid gland | rs2787486 | 0.26 | 0.06 | 1.292 (1.143-1.460) | 4.24E-05 |
| Overall Breast Cancer | Malignant neoplasm of thyroid gland | rs2813549 | 0.26 | 0.06 | 1.295 (1.146-1.463) | 3.30E-05 |
| Overall Breast Cancer | Malignant neoplasm of thyroid gland | rs2822999 | 0.26 | 0.06 | 1.291 (1.142-1.459) | 4.20E-05 |
| Overall Breast Cancer | Malignant neoplasm of thyroid gland | rs2853669 | 0.25 | 0.06 | 1.287 (1.139-1.455) | 5.15E-05 |
| Overall Breast Cancer | Malignant neoplasm of thyroid gland | rs28539243 | 0.26 | 0.06 | 1.298 (1.149-1.466) | 2.66E-05 |
| Overall Breast Cancer | Malignant neoplasm of thyroid gland | rs2886671 | 0.25 | 0.06 | 1.290 (1.142-1.458) | 4.34E-05 |
| Overall Breast Cancer | Malignant neoplasm of thyroid gland | rs2992756 | 0.26 | 0.06 | 1.292 (1.144-1.460) | 3.97E-05 |
| Overall Breast Cancer | Malignant neoplasm of thyroid gland | rs3217992 | 0.26 | 0.06 | 1.291 (1.143-1.459) | 4.18E-05 |
| Overall Breast Cancer | Malignant neoplasm of thyroid gland | rs332529 | 0.26 | 0.06 | 1.293 (1.144-1.461) | 3.72E-05 |
| Overall Breast Cancer | Malignant neoplasm of thyroid gland | rs34005590 | 0.26 | 0.06 | 1.295 (1.146-1.464) | 3.49E-05 |
| Overall Breast Cancer | Malignant neoplasm of thyroid gland | rs35383942 | 0.26 | 0.06 | 1.296 (1.147-1.465) | 3.27E-05 |
| Overall Breast Cancer | Malignant neoplasm of thyroid gland | rs36107432 | 0.25 | 0.06 | 1.290 (1.141-1.457) | 4.44E-05 |
| Overall Breast Cancer | Malignant neoplasm of thyroid gland | rs3741698 | 0.26 | 0.06 | 1.293 (1.145-1.461) | 3.59E-05 |
| Overall Breast Cancer | Malignant neoplasm of thyroid gland | rs3769821 | 0.25 | 0.06 | 1.290 (1.141-1.457) | 4.60E-05 |
| Overall Breast Cancer | Malignant neoplasm of thyroid gland | rs3819405 | 0.26 | 0.06 | 1.292 (1.143-1.460) | 3.97E-05 |
| Overall Breast Cancer | Malignant neoplasm of thyroid gland | rs3821902 | 0.26 | 0.06 | 1.292 (1.143-1.460) | 3.95E-05 |
| Overall Breast Cancer | Malignant neoplasm of thyroid gland | rs4076654 | 0.26 | 0.06 | 1.294 (1.145-1.462) | 3.65E-05 |
| Overall Breast Cancer | Malignant neoplasm of thyroid gland | rs4081859 | 0.26 | 0.06 | 1.292 (1.143-1.460) | 4.01E-05 |
| Overall Breast Cancer | Malignant neoplasm of thyroid gland | rs4233486 | 0.26 | 0.06 | 1.291 (1.143-1.459) | 4.05E-05 |
| Overall Breast Cancer | Malignant neoplasm of thyroid gland | rs4286946 | 0.26 | 0.06 | 1.295 (1.146-1.463) | 3.40E-05 |
| Overall Breast Cancer | Malignant neoplasm of thyroid gland | rs4339481 | 0.26 | 0.06 | 1.293 (1.144-1.461) | 3.85E-05 |
| Overall Breast Cancer | Malignant neoplasm of thyroid gland | rs4378452 | 0.26 | 0.06 | 1.292 (1.143-1.460) | 3.95E-05 |
| Overall Breast Cancer | Malignant neoplasm of thyroid gland | rs4442975 | 0.26 | 0.06 | 1.292 (1.141-1.462) | 5.18E-05 |
| Overall Breast Cancer | Malignant neoplasm of thyroid gland | rs4602255 | 0.26 | 0.06 | 1.292 (1.144-1.460) | 3.91E-05 |
| Overall Breast Cancer | Malignant neoplasm of thyroid gland | rs4808961 | 0.26 | 0.06 | 1.291 (1.142-1.458) | 4.24E-05 |
| Overall Breast Cancer | Malignant neoplasm of thyroid gland | rs4843504 | 0.25 | 0.06 | 1.289 (1.141-1.456) | 4.61E-05 |
| Overall Breast Cancer | Malignant neoplasm of thyroid gland | rs4849879 | 0.26 | 0.06 | 1.292 (1.143-1.460) | 4.10E-05 |
| Overall Breast Cancer | Malignant neoplasm of thyroid gland | rs4885096 | 0.26 | 0.06 | 1.293 (1.145-1.461) | 3.62E-05 |
| Overall Breast Cancer | Malignant neoplasm of thyroid gland | rs4983544 | 0.25 | 0.06 | 1.290 (1.142-1.458) | 4.35E-05 |
| Overall Breast Cancer | Malignant neoplasm of thyroid gland | rs506516 | 0.25 | 0.06 | 1.286 (1.138-1.453) | 5.50E-05 |
| Overall Breast Cancer | Malignant neoplasm of thyroid gland | rs552647 | 0.26 | 0.06 | 1.294 (1.144-1.463) | 4.19E-05 |
| Overall Breast Cancer | Malignant neoplasm of thyroid gland | rs55716112 | 0.25 | 0.06 | 1.290 (1.142-1.458) | 4.34E-05 |
| Overall Breast Cancer | Malignant neoplasm of thyroid gland | rs55760189 | 0.25 | 0.06 | 1.288 (1.140-1.455) | 4.86E-05 |
| Overall Breast Cancer | Malignant neoplasm of thyroid gland | rs56069439 | 0.26 | 0.06 | 1.293 (1.145-1.461) | 3.64E-05 |
| Overall Breast Cancer | Malignant neoplasm of thyroid gland | rs56387622 | 0.26 | 0.06 | 1.292 (1.143-1.460) | 4.02E-05 |
| Overall Breast Cancer | Malignant neoplasm of thyroid gland | rs56681946 | 0.26 | 0.06 | 1.301 (1.152-1.469) | 2.21E-05 |
| Overall Breast Cancer | Malignant neoplasm of thyroid gland | rs58058861 | 0.25 | 0.06 | 1.288 (1.140-1.455) | 4.87E-05 |
| Overall Breast Cancer | Malignant neoplasm of thyroid gland | rs58847541 | 0.25 | 0.06 | 1.289 (1.141-1.456) | 4.66E-05 |
| Overall Breast Cancer | Malignant neoplasm of thyroid gland | rs59867004 | 0.26 | 0.06 | 1.291 (1.142-1.459) | 4.22E-05 |
| Overall Breast Cancer | Malignant neoplasm of thyroid gland | rs5995875 | 0.26 | 0.06 | 1.298 (1.149-1.468) | 2.90E-05 |
| Overall Breast Cancer | Malignant neoplasm of thyroid gland | rs5997390 | 0.25 | 0.06 | 1.283 (1.136-1.449) | 5.94E-05 |
| Overall Breast Cancer | Malignant neoplasm of thyroid gland | rs61373376 | 0.26 | 0.06 | 1.294 (1.146-1.462) | 3.41E-05 |
| Overall Breast Cancer | Malignant neoplasm of thyroid gland | rs61390772 | 0.25 | 0.06 | 1.281 (1.133-1.448) | 7.45E-05 |
| Overall Breast Cancer | Malignant neoplasm of thyroid gland | rs61874140 | 0.26 | 0.06 | 1.296 (1.147-1.465) | 3.18E-05 |
| Overall Breast Cancer | Malignant neoplasm of thyroid gland | rs62048402 | 0.26 | 0.06 | 1.294 (1.145-1.463) | 3.54E-05 |
| Overall Breast Cancer | Malignant neoplasm of thyroid gland | rs62064364 | 0.25 | 0.06 | 1.289 (1.141-1.457) | 4.40E-05 |
| Overall Breast Cancer | Malignant neoplasm of thyroid gland | rs62237573 | 0.21 | 0.06 | 1.236 (1.097-1.392) | 4.82E-04 |
| Overall Breast Cancer | Malignant neoplasm of thyroid gland | rs62390069 | 0.25 | 0.06 | 1.289 (1.141-1.456) | 4.57E-05 |
| Overall Breast Cancer | Malignant neoplasm of thyroid gland | rs62517052 | 0.25 | 0.06 | 1.287 (1.139-1.454) | 4.98E-05 |
| Overall Breast Cancer | Malignant neoplasm of thyroid gland | rs630965 | 0.25 | 0.06 | 1.284 (1.135-1.452) | 6.70E-05 |
| Overall Breast Cancer | Malignant neoplasm of thyroid gland | rs637868 | 0.25 | 0.06 | 1.289 (1.141-1.456) | 4.63E-05 |
| Overall Breast Cancer | Malignant neoplasm of thyroid gland | rs6436017 | 0.26 | 0.06 | 1.291 (1.142-1.459) | 4.21E-05 |
| Overall Breast Cancer | Malignant neoplasm of thyroid gland | rs6440006 | 0.26 | 0.06 | 1.293 (1.144-1.461) | 3.83E-05 |
| Overall Breast Cancer | Malignant neoplasm of thyroid gland | rs6554679 | 0.25 | 0.06 | 1.290 (1.142-1.458) | 4.40E-05 |
| Overall Breast Cancer | Malignant neoplasm of thyroid gland | rs6596100 | 0.25 | 0.06 | 1.288 (1.140-1.455) | 4.73E-05 |
| Overall Breast Cancer | Malignant neoplasm of thyroid gland | rs6597981 | 0.25 | 0.06 | 1.287 (1.139-1.454) | 5.01E-05 |
| Overall Breast Cancer | Malignant neoplasm of thyroid gland | rs6762558 | 0.26 | 0.06 | 1.299 (1.150-1.467) | 2.61E-05 |
| Overall Breast Cancer | Malignant neoplasm of thyroid gland | rs67801543 | 0.25 | 0.06 | 1.289 (1.141-1.456) | 4.58E-05 |
| Overall Breast Cancer | Malignant neoplasm of thyroid gland | rs6805189 | 0.25 | 0.06 | 1.290 (1.142-1.457) | 4.41E-05 |
| Overall Breast Cancer | Malignant neoplasm of thyroid gland | rs68056147 | 0.26 | 0.06 | 1.291 (1.142-1.458) | 4.32E-05 |
| Overall Breast Cancer | Malignant neoplasm of thyroid gland | rs6815814 | 0.25 | 0.06 | 1.286 (1.139-1.452) | 4.98E-05 |
| Overall Breast Cancer | Malignant neoplasm of thyroid gland | rs6860806 | 0.26 | 0.06 | 1.296 (1.147-1.463) | 3.02E-05 |
| Overall Breast Cancer | Malignant neoplasm of thyroid gland | rs6904031 | 0.25 | 0.06 | 1.289 (1.140-1.458) | 5.06E-05 |
| Overall Breast Cancer | Malignant neoplasm of thyroid gland | rs7072776 | 0.26 | 0.06 | 1.295 (1.146-1.463) | 3.46E-05 |
| Overall Breast Cancer | Malignant neoplasm of thyroid gland | rs7149262 | 0.26 | 0.06 | 1.294 (1.145-1.463) | 3.55E-05 |
| Overall Breast Cancer | Malignant neoplasm of thyroid gland | rs71559437 | 0.26 | 0.06 | 1.291 (1.143-1.459) | 4.06E-05 |
| Overall Breast Cancer | Malignant neoplasm of thyroid gland | rs7184573 | 0.26 | 0.06 | 1.296 (1.148-1.464) | 2.96E-05 |
| Overall Breast Cancer | Malignant neoplasm of thyroid gland | rs719338 | 0.25 | 0.06 | 1.287 (1.138-1.454) | 5.50E-05 |
| Overall Breast Cancer | Malignant neoplasm of thyroid gland | rs7240205 | 0.26 | 0.06 | 1.291 (1.143-1.459) | 4.08E-05 |
| Overall Breast Cancer | Malignant neoplasm of thyroid gland | rs72658071 | 0.26 | 0.06 | 1.292 (1.143-1.460) | 4.07E-05 |
| Overall Breast Cancer | Malignant neoplasm of thyroid gland | rs72749841 | 0.26 | 0.06 | 1.295 (1.146-1.463) | 3.31E-05 |
| Overall Breast Cancer | Malignant neoplasm of thyroid gland | rs72755295 | 0.25 | 0.06 | 1.287 (1.140-1.454) | 4.92E-05 |
| Overall Breast Cancer | Malignant neoplasm of thyroid gland | rs72832370 | 0.25 | 0.06 | 1.287 (1.139-1.454) | 5.14E-05 |
| Overall Breast Cancer | Malignant neoplasm of thyroid gland | rs72832402 | 0.26 | 0.06 | 1.294 (1.145-1.462) | 3.65E-05 |
| Overall Breast Cancer | Malignant neoplasm of thyroid gland | rs72931898 | 0.26 | 0.06 | 1.292 (1.143-1.460) | 4.09E-05 |
| Overall Breast Cancer | Malignant neoplasm of thyroid gland | rs7297051 | 0.26 | 0.06 | 1.295 (1.145-1.465) | 3.88E-05 |
| Overall Breast Cancer | Malignant neoplasm of thyroid gland | rs73161324 | 0.26 | 0.06 | 1.294 (1.145-1.462) | 3.49E-05 |
| Overall Breast Cancer | Malignant neoplasm of thyroid gland | rs7378815 | 0.25 | 0.06 | 1.287 (1.139-1.453) | 4.93E-05 |
| Overall Breast Cancer | Malignant neoplasm of thyroid gland | rs738321 | 0.26 | 0.06 | 1.294 (1.145-1.462) | 3.50E-05 |
| Overall Breast Cancer | Malignant neoplasm of thyroid gland | rs7463114 | 0.26 | 0.06 | 1.295 (1.146-1.463) | 3.47E-05 |
| Overall Breast Cancer | Malignant neoplasm of thyroid gland | rs7499149 | 0.25 | 0.06 | 1.287 (1.139-1.455) | 5.44E-05 |
| Overall Breast Cancer | Malignant neoplasm of thyroid gland | rs7513707 | 0.25 | 0.06 | 1.289 (1.140-1.456) | 4.72E-05 |
| Overall Breast Cancer | Malignant neoplasm of thyroid gland | rs7529522 | 0.25 | 0.06 | 1.286 (1.139-1.453) | 5.07E-05 |
| Overall Breast Cancer | Malignant neoplasm of thyroid gland | rs7697216 | 0.25 | 0.06 | 1.285 (1.137-1.452) | 5.96E-05 |
| Overall Breast Cancer | Malignant neoplasm of thyroid gland | rs7736 | 0.26 | 0.06 | 1.292 (1.144-1.460) | 3.85E-05 |
| Overall Breast Cancer | Malignant neoplasm of thyroid gland | rs77528541 | 0.26 | 0.06 | 1.297 (1.148-1.464) | 2.77E-05 |
| Overall Breast Cancer | Malignant neoplasm of thyroid gland | rs7760611 | 0.26 | 0.06 | 1.292 (1.144-1.460) | 3.83E-05 |
| Overall Breast Cancer | Malignant neoplasm of thyroid gland | rs78269692 | 0.26 | 0.06 | 1.297 (1.149-1.465) | 2.66E-05 |
| Overall Breast Cancer | Malignant neoplasm of thyroid gland | rs78440108 | 0.26 | 0.06 | 1.292 (1.143-1.459) | 4.09E-05 |
| Overall Breast Cancer | Malignant neoplasm of thyroid gland | rs78540526 | 0.28 | 0.06 | 1.320 (1.166-1.494) | 1.13E-05 |
| Overall Breast Cancer | Malignant neoplasm of thyroid gland | rs7939702 | 0.25 | 0.06 | 1.288 (1.140-1.456) | 4.70E-05 |
| Overall Breast Cancer | Malignant neoplasm of thyroid gland | rs7971 | 0.25 | 0.06 | 1.286 (1.139-1.453) | 4.89E-05 |
| Overall Breast Cancer | Malignant neoplasm of thyroid gland | rs79724016 | 0.25 | 0.06 | 1.289 (1.141-1.456) | 4.65E-05 |
| Overall Breast Cancer | Malignant neoplasm of thyroid gland | rs8105994 | 0.26 | 0.06 | 1.292 (1.143-1.460) | 4.16E-05 |
| Overall Breast Cancer | Malignant neoplasm of thyroid gland | rs889310 | 0.26 | 0.06 | 1.291 (1.142-1.458) | 4.24E-05 |
| Overall Breast Cancer | Malignant neoplasm of thyroid gland | rs941764 | 0.25 | 0.06 | 1.290 (1.141-1.457) | 4.52E-05 |
| Overall Breast Cancer | Malignant neoplasm of thyroid gland | rs9611271 | 0.26 | 0.06 | 1.298 (1.150-1.465) | 2.47E-05 |
| Overall Breast Cancer | Malignant neoplasm of thyroid gland | rs9620778 | 0.25 | 0.06 | 1.284 (1.137-1.449) | 5.65E-05 |
| Overall Breast Cancer | Malignant neoplasm of thyroid gland | rs970822 | 0.25 | 0.06 | 1.287 (1.139-1.454) | 4.94E-05 |
| Overall Breast Cancer | Malignant neoplasm of thyroid gland | rs9712235 | 0.26 | 0.06 | 1.294 (1.145-1.462) | 3.46E-05 |
| Overall Breast Cancer | Malignant neoplasm of thyroid gland | rs9833888 | 0.25 | 0.06 | 1.289 (1.141-1.456) | 4.68E-05 |
| Overall Breast Cancer | Malignant neoplasm of thyroid gland | rs9952980 | 0.25 | 0.06 | 1.288 (1.140-1.456) | 4.79E-05 |
| Overall Breast Cancer | Malignant neoplasm of thyroid gland | rs9954058 | 0.26 | 0.06 | 1.293 (1.145-1.461) | 3.66E-05 |
| Overall Breast Cancer | Malignant neoplasm of thyroid gland | All | 0.26 | 0.06 | 1.291 (1.143-1.458) | 3.85E-05 |

**Table S7. The result of leave-one-out approach for Overall Breast Cancer and Benign neoplasm of thyroid gland**

| **Exposure** | **Outcome** | **SNP** | **Beta** | **Standard error** | **OR (95%CI)** | **P-value** |
| --- | --- | --- | --- | --- | --- | --- |
| Overall Breast Cancer | Benign neoplasm of thyroid gland | rs10022462 | 0.05 | 0.07 | 1.052 (0.923-1.199) | 4.50E-01 |
| Overall Breast Cancer | Benign neoplasm of thyroid gland | rs10074269 | 0.05 | 0.07 | 1.048 (0.920-1.193) | 4.84E-01 |
| Overall Breast Cancer | Benign neoplasm of thyroid gland | rs10096351 | 0.04 | 0.07 | 1.043 (0.914-1.190) | 5.29E-01 |
| Overall Breast Cancer | Benign neoplasm of thyroid gland | rs1016578 | 0.05 | 0.07 | 1.049 (0.921-1.196) | 4.71E-01 |
| Overall Breast Cancer | Benign neoplasm of thyroid gland | rs10760444 | 0.05 | 0.07 | 1.052 (0.923-1.199) | 4.48E-01 |
| Overall Breast Cancer | Benign neoplasm of thyroid gland | rs10762849 | 0.05 | 0.07 | 1.052 (0.922-1.199) | 4.53E-01 |
| Overall Breast Cancer | Benign neoplasm of thyroid gland | rs10816625 | 0.05 | 0.07 | 1.055 (0.925-1.202) | 4.27E-01 |
| Overall Breast Cancer | Benign neoplasm of thyroid gland | rs10838267 | 0.05 | 0.07 | 1.048 (0.920-1.194) | 4.79E-01 |
| Overall Breast Cancer | Benign neoplasm of thyroid gland | rs10885405 | 0.05 | 0.07 | 1.056 (0.926-1.203) | 4.16E-01 |
| Overall Breast Cancer | Benign neoplasm of thyroid gland | rs10941679 | 0.07 | 0.07 | 1.071 (0.939-1.221) | 3.06E-01 |
| Overall Breast Cancer | Benign neoplasm of thyroid gland | rs10941712 | 0.05 | 0.07 | 1.052 (0.923-1.199) | 4.50E-01 |
| Overall Breast Cancer | Benign neoplasm of thyroid gland | rs10978911 | 0.05 | 0.07 | 1.050 (0.921-1.198) | 4.62E-01 |
| Overall Breast Cancer | Benign neoplasm of thyroid gland | rs11049420 | 0.05 | 0.07 | 1.056 (0.926-1.203) | 4.18E-01 |
| Overall Breast Cancer | Benign neoplasm of thyroid gland | rs11116495 | 0.06 | 0.07 | 1.058 (0.930-1.205) | 3.91E-01 |
| Overall Breast Cancer | Benign neoplasm of thyroid gland | rs11117758 | 0.05 | 0.07 | 1.050 (0.921-1.197) | 4.63E-01 |
| Overall Breast Cancer | Benign neoplasm of thyroid gland | rs11205303 | 0.05 | 0.07 | 1.051 (0.922-1.199) | 4.53E-01 |
| Overall Breast Cancer | Benign neoplasm of thyroid gland | rs11249433 | 0.05 | 0.07 | 1.051 (0.922-1.200) | 4.55E-01 |
| Overall Breast Cancer | Benign neoplasm of thyroid gland | rs11264454 | 0.05 | 0.07 | 1.052 (0.923-1.199) | 4.48E-01 |
| Overall Breast Cancer | Benign neoplasm of thyroid gland | rs113577745 | 0.05 | 0.07 | 1.051 (0.922-1.198) | 4.53E-01 |
| Overall Breast Cancer | Benign neoplasm of thyroid gland | rs11571833 | 0.05 | 0.07 | 1.056 (0.926-1.204) | 4.15E-01 |
| Overall Breast Cancer | Benign neoplasm of thyroid gland | rs11583393 | 0.04 | 0.07 | 1.046 (0.919-1.191) | 4.99E-01 |
| Overall Breast Cancer | Benign neoplasm of thyroid gland | rs11624333 | 0.06 | 0.07 | 1.059 (0.928-1.207) | 3.95E-01 |
| Overall Breast Cancer | Benign neoplasm of thyroid gland | rs11652463 | 0.05 | 0.07 | 1.052 (0.923-1.199) | 4.48E-01 |
| Overall Breast Cancer | Benign neoplasm of thyroid gland | rs11680449 | 0.05 | 0.07 | 1.052 (0.923-1.199) | 4.46E-01 |
| Overall Breast Cancer | Benign neoplasm of thyroid gland | rs11684853 | 0.05 | 0.07 | 1.050 (0.922-1.197) | 4.62E-01 |
| Overall Breast Cancer | Benign neoplasm of thyroid gland | rs11693806 | 0.05 | 0.07 | 1.051 (0.922-1.199) | 4.54E-01 |
| Overall Breast Cancer | Benign neoplasm of thyroid gland | rs11749176 | 0.05 | 0.07 | 1.050 (0.922-1.197) | 4.63E-01 |
| Overall Breast Cancer | Benign neoplasm of thyroid gland | rs11822830 | 0.05 | 0.07 | 1.052 (0.923-1.199) | 4.48E-01 |
| Overall Breast Cancer | Benign neoplasm of thyroid gland | rs11903787 | 0.05 | 0.07 | 1.054 (0.925-1.201) | 4.32E-01 |
| Overall Breast Cancer | Benign neoplasm of thyroid gland | rs11977670 | 0.06 | 0.07 | 1.059 (0.930-1.207) | 3.86E-01 |
| Overall Breast Cancer | Benign neoplasm of thyroid gland | rs12091730 | 0.05 | 0.07 | 1.056 (0.926-1.203) | 4.16E-01 |
| Overall Breast Cancer | Benign neoplasm of thyroid gland | rs12250948 | 0.05 | 0.07 | 1.050 (0.921-1.196) | 4.69E-01 |
| Overall Breast Cancer | Benign neoplasm of thyroid gland | rs12260388 | 0.05 | 0.07 | 1.048 (0.920-1.194) | 4.79E-01 |
| Overall Breast Cancer | Benign neoplasm of thyroid gland | rs12422552 | 0.06 | 0.07 | 1.057 (0.928-1.205) | 4.01E-01 |
| Overall Breast Cancer | Benign neoplasm of thyroid gland | rs12479355 | 0.05 | 0.07 | 1.054 (0.924-1.201) | 4.34E-01 |
| Overall Breast Cancer | Benign neoplasm of thyroid gland | rs12493607 | 0.05 | 0.07 | 1.056 (0.927-1.204) | 4.11E-01 |
| Overall Breast Cancer | Benign neoplasm of thyroid gland | rs12519859 | 0.06 | 0.07 | 1.060 (0.931-1.205) | 3.80E-01 |
| Overall Breast Cancer | Benign neoplasm of thyroid gland | rs12594752 | 0.06 | 0.07 | 1.062 (0.933-1.209) | 3.62E-01 |
| Overall Breast Cancer | Benign neoplasm of thyroid gland | rs12628403 | 0.05 | 0.07 | 1.052 (0.923-1.200) | 4.44E-01 |
| Overall Breast Cancer | Benign neoplasm of thyroid gland | rs12652713 | 0.05 | 0.07 | 1.053 (0.924-1.200) | 4.41E-01 |
| Overall Breast Cancer | Benign neoplasm of thyroid gland | rs12653202 | 0.06 | 0.07 | 1.058 (0.927-1.207) | 4.06E-01 |
| Overall Breast Cancer | Benign neoplasm of thyroid gland | rs12706954 | 0.05 | 0.07 | 1.047 (0.919-1.193) | 4.89E-01 |
| Overall Breast Cancer | Benign neoplasm of thyroid gland | rs12765365 | 0.05 | 0.07 | 1.048 (0.920-1.193) | 4.83E-01 |
| Overall Breast Cancer | Benign neoplasm of thyroid gland | rs12894297 | 0.05 | 0.07 | 1.053 (0.924-1.200) | 4.40E-01 |
| Overall Breast Cancer | Benign neoplasm of thyroid gland | rs12962334 | 0.05 | 0.07 | 1.054 (0.925-1.201) | 4.32E-01 |
| Overall Breast Cancer | Benign neoplasm of thyroid gland | rs13039563 | 0.06 | 0.07 | 1.057 (0.928-1.204) | 4.07E-01 |
| Overall Breast Cancer | Benign neoplasm of thyroid gland | rs13066793 | 0.05 | 0.07 | 1.052 (0.923-1.199) | 4.48E-01 |
| Overall Breast Cancer | Benign neoplasm of thyroid gland | rs132365 | 0.05 | 0.07 | 1.048 (0.919-1.194) | 4.87E-01 |
| Overall Breast Cancer | Benign neoplasm of thyroid gland | rs13256025 | 0.05 | 0.07 | 1.053 (0.924-1.200) | 4.40E-01 |
| Overall Breast Cancer | Benign neoplasm of thyroid gland | rs13267382 | 0.05 | 0.07 | 1.052 (0.923-1.199) | 4.51E-01 |
| Overall Breast Cancer | Benign neoplasm of thyroid gland | rs13277568 | 0.05 | 0.07 | 1.056 (0.927-1.203) | 4.14E-01 |
| Overall Breast Cancer | Benign neoplasm of thyroid gland | rs1361549 | 0.05 | 0.07 | 1.053 (0.924-1.201) | 4.37E-01 |
| Overall Breast Cancer | Benign neoplasm of thyroid gland | rs1375631 | 0.05 | 0.07 | 1.049 (0.921-1.195) | 4.72E-01 |
| Overall Breast Cancer | Benign neoplasm of thyroid gland | rs139331653 | 0.05 | 0.07 | 1.047 (0.919-1.193) | 4.87E-01 |
| Overall Breast Cancer | Benign neoplasm of thyroid gland | rs140091 | 0.05 | 0.07 | 1.049 (0.921-1.196) | 4.70E-01 |
| Overall Breast Cancer | Benign neoplasm of thyroid gland | rs141930488 | 0.05 | 0.07 | 1.055 (0.926-1.202) | 4.20E-01 |
| Overall Breast Cancer | Benign neoplasm of thyroid gland | rs1467576 | 0.05 | 0.07 | 1.051 (0.922-1.198) | 4.56E-01 |
| Overall Breast Cancer | Benign neoplasm of thyroid gland | rs148893083 | 0.04 | 0.07 | 1.044 (0.918-1.188) | 5.09E-01 |
| Overall Breast Cancer | Benign neoplasm of thyroid gland | rs150540840 | 0.05 | 0.07 | 1.047 (0.919-1.194) | 4.90E-01 |
| Overall Breast Cancer | Benign neoplasm of thyroid gland | rs1511243 | 0.06 | 0.07 | 1.058 (0.928-1.206) | 3.99E-01 |
| Overall Breast Cancer | Benign neoplasm of thyroid gland | rs1531212 | 0.05 | 0.07 | 1.056 (0.927-1.204) | 4.09E-01 |
| Overall Breast Cancer | Benign neoplasm of thyroid gland | rs1533366 | 0.06 | 0.07 | 1.062 (0.934-1.208) | 3.60E-01 |
| Overall Breast Cancer | Benign neoplasm of thyroid gland | rs1541409 | 0.05 | 0.07 | 1.051 (0.922-1.197) | 4.59E-01 |
| Overall Breast Cancer | Benign neoplasm of thyroid gland | rs16991615 | 0.05 | 0.07 | 1.052 (0.923-1.199) | 4.44E-01 |
| Overall Breast Cancer | Benign neoplasm of thyroid gland | rs1707302 | 0.05 | 0.07 | 1.048 (0.920-1.194) | 4.82E-01 |
| Overall Breast Cancer | Benign neoplasm of thyroid gland | rs170801 | 0.05 | 0.07 | 1.052 (0.923-1.199) | 4.50E-01 |
| Overall Breast Cancer | Benign neoplasm of thyroid gland | rs17181761 | 0.05 | 0.07 | 1.051 (0.922-1.197) | 4.60E-01 |
| Overall Breast Cancer | Benign neoplasm of thyroid gland | rs17271951 | 0.04 | 0.07 | 1.043 (0.912-1.194) | 5.37E-01 |
| Overall Breast Cancer | Benign neoplasm of thyroid gland | rs17356907 | 0.07 | 0.07 | 1.067 (0.937-1.216) | 3.26E-01 |
| Overall Breast Cancer | Benign neoplasm of thyroid gland | rs17732378 | 0.05 | 0.07 | 1.049 (0.921-1.195) | 4.73E-01 |
| Overall Breast Cancer | Benign neoplasm of thyroid gland | rs1836962 | 0.06 | 0.07 | 1.057 (0.928-1.204) | 4.07E-01 |
| Overall Breast Cancer | Benign neoplasm of thyroid gland | rs1895062 | 0.05 | 0.07 | 1.053 (0.924-1.200) | 4.40E-01 |
| Overall Breast Cancer | Benign neoplasm of thyroid gland | rs1909666 | 0.05 | 0.07 | 1.054 (0.925-1.201) | 4.31E-01 |
| Overall Breast Cancer | Benign neoplasm of thyroid gland | rs1973765 | 0.06 | 0.07 | 1.059 (0.929-1.208) | 3.90E-01 |
| Overall Breast Cancer | Benign neoplasm of thyroid gland | rs2010610 | 0.05 | 0.07 | 1.053 (0.924-1.200) | 4.41E-01 |
| Overall Breast Cancer | Benign neoplasm of thyroid gland | rs2016394 | 0.05 | 0.07 | 1.047 (0.919-1.193) | 4.89E-01 |
| Overall Breast Cancer | Benign neoplasm of thyroid gland | rs2028195 | 0.05 | 0.07 | 1.054 (0.925-1.202) | 4.27E-01 |
| Overall Breast Cancer | Benign neoplasm of thyroid gland | rs2075881 | 0.05 | 0.07 | 1.056 (0.927-1.203) | 4.15E-01 |
| Overall Breast Cancer | Benign neoplasm of thyroid gland | rs2121348 | 0.05 | 0.07 | 1.056 (0.927-1.203) | 4.11E-01 |
| Overall Breast Cancer | Benign neoplasm of thyroid gland | rs2141818 | 0.06 | 0.07 | 1.058 (0.928-1.205) | 3.98E-01 |
| Overall Breast Cancer | Benign neoplasm of thyroid gland | rs2188648 | 0.05 | 0.07 | 1.055 (0.926-1.203) | 4.20E-01 |
| Overall Breast Cancer | Benign neoplasm of thyroid gland | rs2223621 | 0.05 | 0.07 | 1.049 (0.920-1.195) | 4.76E-01 |
| Overall Breast Cancer | Benign neoplasm of thyroid gland | rs2253012 | 0.05 | 0.07 | 1.052 (0.923-1.200) | 4.44E-01 |
| Overall Breast Cancer | Benign neoplasm of thyroid gland | rs2403907 | 0.05 | 0.07 | 1.053 (0.923-1.201) | 4.41E-01 |
| Overall Breast Cancer | Benign neoplasm of thyroid gland | rs2408652 | 0.05 | 0.07 | 1.050 (0.921-1.197) | 4.66E-01 |
| Overall Breast Cancer | Benign neoplasm of thyroid gland | rs2420941 | 0.05 | 0.07 | 1.048 (0.920-1.193) | 4.83E-01 |
| Overall Breast Cancer | Benign neoplasm of thyroid gland | rs2454399 | 0.04 | 0.07 | 1.041 (0.913-1.186) | 5.47E-01 |
| Overall Breast Cancer | Benign neoplasm of thyroid gland | rs2601774 | 0.05 | 0.07 | 1.051 (0.922-1.198) | 4.56E-01 |
| Overall Breast Cancer | Benign neoplasm of thyroid gland | rs2787486 | 0.05 | 0.07 | 1.047 (0.918-1.193) | 4.93E-01 |
| Overall Breast Cancer | Benign neoplasm of thyroid gland | rs2813549 | 0.05 | 0.07 | 1.051 (0.922-1.198) | 4.55E-01 |
| Overall Breast Cancer | Benign neoplasm of thyroid gland | rs2822999 | 0.05 | 0.07 | 1.056 (0.927-1.204) | 4.11E-01 |
| Overall Breast Cancer | Benign neoplasm of thyroid gland | rs2853669 | 0.05 | 0.07 | 1.050 (0.921-1.197) | 4.68E-01 |
| Overall Breast Cancer | Benign neoplasm of thyroid gland | rs28539243 | 0.04 | 0.07 | 1.041 (0.915-1.184) | 5.39E-01 |
| Overall Breast Cancer | Benign neoplasm of thyroid gland | rs2886671 | 0.05 | 0.07 | 1.049 (0.921-1.195) | 4.73E-01 |
| Overall Breast Cancer | Benign neoplasm of thyroid gland | rs2992756 | 0.05 | 0.07 | 1.049 (0.920-1.196) | 4.72E-01 |
| Overall Breast Cancer | Benign neoplasm of thyroid gland | rs3217992 | 0.05 | 0.07 | 1.053 (0.924-1.201) | 4.38E-01 |
| Overall Breast Cancer | Benign neoplasm of thyroid gland | rs332529 | 0.05 | 0.07 | 1.051 (0.922-1.198) | 4.56E-01 |
| Overall Breast Cancer | Benign neoplasm of thyroid gland | rs34005590 | 0.06 | 0.07 | 1.066 (0.936-1.214) | 3.36E-01 |
| Overall Breast Cancer | Benign neoplasm of thyroid gland | rs35383942 | 0.06 | 0.07 | 1.061 (0.931-1.209) | 3.74E-01 |
| Overall Breast Cancer | Benign neoplasm of thyroid gland | rs36107432 | 0.05 | 0.07 | 1.053 (0.924-1.201) | 4.37E-01 |
| Overall Breast Cancer | Benign neoplasm of thyroid gland | rs3741698 | 0.05 | 0.07 | 1.053 (0.924-1.200) | 4.37E-01 |
| Overall Breast Cancer | Benign neoplasm of thyroid gland | rs3769821 | 0.05 | 0.07 | 1.048 (0.920-1.195) | 4.80E-01 |
| Overall Breast Cancer | Benign neoplasm of thyroid gland | rs3819405 | 0.05 | 0.07 | 1.051 (0.922-1.199) | 4.52E-01 |
| Overall Breast Cancer | Benign neoplasm of thyroid gland | rs3821902 | 0.05 | 0.07 | 1.053 (0.924-1.201) | 4.36E-01 |
| Overall Breast Cancer | Benign neoplasm of thyroid gland | rs4076654 | 0.05 | 0.07 | 1.051 (0.922-1.198) | 4.60E-01 |
| Overall Breast Cancer | Benign neoplasm of thyroid gland | rs4081859 | 0.05 | 0.07 | 1.056 (0.927-1.204) | 4.10E-01 |
| Overall Breast Cancer | Benign neoplasm of thyroid gland | rs4233486 | 0.05 | 0.07 | 1.048 (0.920-1.194) | 4.78E-01 |
| Overall Breast Cancer | Benign neoplasm of thyroid gland | rs4286946 | 0.05 | 0.07 | 1.051 (0.922-1.199) | 4.54E-01 |
| Overall Breast Cancer | Benign neoplasm of thyroid gland | rs4339481 | 0.05 | 0.07 | 1.056 (0.927-1.204) | 4.11E-01 |
| Overall Breast Cancer | Benign neoplasm of thyroid gland | rs4378452 | 0.05 | 0.07 | 1.052 (0.923-1.200) | 4.44E-01 |
| Overall Breast Cancer | Benign neoplasm of thyroid gland | rs4442975 | 0.04 | 0.07 | 1.044 (0.914-1.192) | 5.26E-01 |
| Overall Breast Cancer | Benign neoplasm of thyroid gland | rs4602255 | 0.05 | 0.07 | 1.050 (0.921-1.197) | 4.65E-01 |
| Overall Breast Cancer | Benign neoplasm of thyroid gland | rs4808961 | 0.05 | 0.07 | 1.049 (0.921-1.195) | 4.73E-01 |
| Overall Breast Cancer | Benign neoplasm of thyroid gland | rs4843504 | 0.05 | 0.07 | 1.056 (0.927-1.203) | 4.16E-01 |
| Overall Breast Cancer | Benign neoplasm of thyroid gland | rs4849879 | 0.04 | 0.07 | 1.046 (0.918-1.192) | 5.00E-01 |
| Overall Breast Cancer | Benign neoplasm of thyroid gland | rs4885096 | 0.05 | 0.07 | 1.050 (0.921-1.196) | 4.66E-01 |
| Overall Breast Cancer | Benign neoplasm of thyroid gland | rs4983544 | 0.05 | 0.07 | 1.048 (0.920-1.194) | 4.81E-01 |
| Overall Breast Cancer | Benign neoplasm of thyroid gland | rs506516 | 0.05 | 0.07 | 1.046 (0.918-1.192) | 4.98E-01 |
| Overall Breast Cancer | Benign neoplasm of thyroid gland | rs552647 | 0.07 | 0.07 | 1.067 (0.936-1.217) | 3.31E-01 |
| Overall Breast Cancer | Benign neoplasm of thyroid gland | rs55716112 | 0.05 | 0.07 | 1.051 (0.922-1.198) | 4.53E-01 |
| Overall Breast Cancer | Benign neoplasm of thyroid gland | rs55760189 | 0.05 | 0.07 | 1.056 (0.927-1.203) | 4.14E-01 |
| Overall Breast Cancer | Benign neoplasm of thyroid gland | rs56069439 | 0.05 | 0.07 | 1.050 (0.921-1.197) | 4.64E-01 |
| Overall Breast Cancer | Benign neoplasm of thyroid gland | rs56387622 | 0.04 | 0.07 | 1.046 (0.918-1.192) | 5.02E-01 |
| Overall Breast Cancer | Benign neoplasm of thyroid gland | rs56681946 | 0.05 | 0.07 | 1.055 (0.926-1.203) | 4.21E-01 |
| Overall Breast Cancer | Benign neoplasm of thyroid gland | rs58058861 | 0.05 | 0.07 | 1.053 (0.924-1.201) | 4.35E-01 |
| Overall Breast Cancer | Benign neoplasm of thyroid gland | rs58847541 | 0.05 | 0.07 | 1.049 (0.921-1.196) | 4.71E-01 |
| Overall Breast Cancer | Benign neoplasm of thyroid gland | rs59867004 | 0.05 | 0.07 | 1.049 (0.921-1.196) | 4.73E-01 |
| Overall Breast Cancer | Benign neoplasm of thyroid gland | rs5995875 | 0.05 | 0.07 | 1.051 (0.921-1.198) | 4.60E-01 |
| Overall Breast Cancer | Benign neoplasm of thyroid gland | rs5997390 | 0.05 | 0.07 | 1.053 (0.924-1.201) | 4.39E-01 |
| Overall Breast Cancer | Benign neoplasm of thyroid gland | rs61373376 | 0.05 | 0.07 | 1.052 (0.922-1.199) | 4.52E-01 |
| Overall Breast Cancer | Benign neoplasm of thyroid gland | rs61390772 | 0.06 | 0.07 | 1.058 (0.927-1.206) | 4.04E-01 |
| Overall Breast Cancer | Benign neoplasm of thyroid gland | rs61874140 | 0.05 | 0.07 | 1.053 (0.923-1.200) | 4.41E-01 |
| Overall Breast Cancer | Benign neoplasm of thyroid gland | rs62048402 | 0.05 | 0.07 | 1.055 (0.925-1.202) | 4.25E-01 |
| Overall Breast Cancer | Benign neoplasm of thyroid gland | rs62064364 | 0.05 | 0.07 | 1.054 (0.925-1.201) | 4.32E-01 |
| Overall Breast Cancer | Benign neoplasm of thyroid gland | rs62237573 | 0.04 | 0.07 | 1.043 (0.914-1.191) | 5.28E-01 |
| Overall Breast Cancer | Benign neoplasm of thyroid gland | rs62390069 | 0.06 | 0.07 | 1.061 (0.933-1.206) | 3.66E-01 |
| Overall Breast Cancer | Benign neoplasm of thyroid gland | rs62517052 | 0.05 | 0.07 | 1.049 (0.921-1.196) | 4.71E-01 |
| Overall Breast Cancer | Benign neoplasm of thyroid gland | rs630965 | 0.04 | 0.07 | 1.039 (0.911-1.184) | 5.70E-01 |
| Overall Breast Cancer | Benign neoplasm of thyroid gland | rs637868 | 0.05 | 0.07 | 1.055 (0.926-1.203) | 4.19E-01 |
| Overall Breast Cancer | Benign neoplasm of thyroid gland | rs6436017 | 0.05 | 0.07 | 1.047 (0.919-1.193) | 4.89E-01 |
| Overall Breast Cancer | Benign neoplasm of thyroid gland | rs6440006 | 0.05 | 0.07 | 1.052 (0.923-1.199) | 4.48E-01 |
| Overall Breast Cancer | Benign neoplasm of thyroid gland | rs6554679 | 0.05 | 0.07 | 1.053 (0.924-1.201) | 4.36E-01 |
| Overall Breast Cancer | Benign neoplasm of thyroid gland | rs6596100 | 0.05 | 0.07 | 1.056 (0.927-1.203) | 4.15E-01 |
| Overall Breast Cancer | Benign neoplasm of thyroid gland | rs6597981 | 0.05 | 0.07 | 1.051 (0.922-1.198) | 4.59E-01 |
| Overall Breast Cancer | Benign neoplasm of thyroid gland | rs6762558 | 0.05 | 0.07 | 1.051 (0.922-1.198) | 4.57E-01 |
| Overall Breast Cancer | Benign neoplasm of thyroid gland | rs67801543 | 0.05 | 0.07 | 1.051 (0.922-1.197) | 4.60E-01 |
| Overall Breast Cancer | Benign neoplasm of thyroid gland | rs6805189 | 0.05 | 0.07 | 1.053 (0.923-1.200) | 4.43E-01 |
| Overall Breast Cancer | Benign neoplasm of thyroid gland | rs68056147 | 0.06 | 0.07 | 1.058 (0.928-1.205) | 3.98E-01 |
| Overall Breast Cancer | Benign neoplasm of thyroid gland | rs6815814 | 0.05 | 0.07 | 1.056 (0.927-1.203) | 4.11E-01 |
| Overall Breast Cancer | Benign neoplasm of thyroid gland | rs6860806 | 0.05 | 0.07 | 1.056 (0.926-1.203) | 4.16E-01 |
| Overall Breast Cancer | Benign neoplasm of thyroid gland | rs6904031 | 0.05 | 0.07 | 1.052 (0.922-1.200) | 4.48E-01 |
| Overall Breast Cancer | Benign neoplasm of thyroid gland | rs7072776 | 0.05 | 0.07 | 1.050 (0.921-1.197) | 4.64E-01 |
| Overall Breast Cancer | Benign neoplasm of thyroid gland | rs7149262 | 0.06 | 0.07 | 1.067 (0.938-1.213) | 3.23E-01 |
| Overall Breast Cancer | Benign neoplasm of thyroid gland | rs71559437 | 0.05 | 0.07 | 1.052 (0.923-1.199) | 4.49E-01 |
| Overall Breast Cancer | Benign neoplasm of thyroid gland | rs7184573 | 0.05 | 0.07 | 1.050 (0.922-1.197) | 4.62E-01 |
| Overall Breast Cancer | Benign neoplasm of thyroid gland | rs719338 | 0.06 | 0.07 | 1.059 (0.929-1.208) | 3.89E-01 |
| Overall Breast Cancer | Benign neoplasm of thyroid gland | rs7240205 | 0.06 | 0.07 | 1.058 (0.929-1.205) | 3.96E-01 |
| Overall Breast Cancer | Benign neoplasm of thyroid gland | rs72658071 | 0.04 | 0.07 | 1.043 (0.917-1.187) | 5.21E-01 |
| Overall Breast Cancer | Benign neoplasm of thyroid gland | rs72749841 | 0.05 | 0.07 | 1.048 (0.920-1.195) | 4.78E-01 |
| Overall Breast Cancer | Benign neoplasm of thyroid gland | rs72755295 | 0.05 | 0.07 | 1.055 (0.925-1.202) | 4.26E-01 |
| Overall Breast Cancer | Benign neoplasm of thyroid gland | rs72832370 | 0.05 | 0.07 | 1.054 (0.925-1.202) | 4.27E-01 |
| Overall Breast Cancer | Benign neoplasm of thyroid gland | rs72832402 | 0.05 | 0.07 | 1.052 (0.923-1.199) | 4.51E-01 |
| Overall Breast Cancer | Benign neoplasm of thyroid gland | rs72931898 | 0.05 | 0.07 | 1.052 (0.923-1.200) | 4.46E-01 |
| Overall Breast Cancer | Benign neoplasm of thyroid gland | rs7297051 | 0.05 | 0.07 | 1.047 (0.917-1.194) | 4.99E-01 |
| Overall Breast Cancer | Benign neoplasm of thyroid gland | rs73161324 | 0.05 | 0.07 | 1.050 (0.921-1.197) | 4.64E-01 |
| Overall Breast Cancer | Benign neoplasm of thyroid gland | rs7378815 | 0.05 | 0.07 | 1.055 (0.926-1.202) | 4.24E-01 |
| Overall Breast Cancer | Benign neoplasm of thyroid gland | rs738321 | 0.05 | 0.07 | 1.047 (0.919-1.193) | 4.90E-01 |
| Overall Breast Cancer | Benign neoplasm of thyroid gland | rs7463114 | 0.05 | 0.07 | 1.052 (0.923-1.200) | 4.44E-01 |
| Overall Breast Cancer | Benign neoplasm of thyroid gland | rs7499149 | 0.06 | 0.07 | 1.058 (0.928-1.207) | 3.96E-01 |
| Overall Breast Cancer | Benign neoplasm of thyroid gland | rs7513707 | 0.04 | 0.07 | 1.046 (0.918-1.191) | 5.01E-01 |
| Overall Breast Cancer | Benign neoplasm of thyroid gland | rs7529522 | 0.05 | 0.07 | 1.054 (0.925-1.202) | 4.28E-01 |
| Overall Breast Cancer | Benign neoplasm of thyroid gland | rs7697216 | 0.06 | 0.07 | 1.061 (0.931-1.209) | 3.76E-01 |
| Overall Breast Cancer | Benign neoplasm of thyroid gland | rs7736 | 0.05 | 0.07 | 1.053 (0.924-1.200) | 4.36E-01 |
| Overall Breast Cancer | Benign neoplasm of thyroid gland | rs77528541 | 0.04 | 0.07 | 1.046 (0.919-1.191) | 4.97E-01 |
| Overall Breast Cancer | Benign neoplasm of thyroid gland | rs7760611 | 0.05 | 0.07 | 1.048 (0.920-1.194) | 4.82E-01 |
| Overall Breast Cancer | Benign neoplasm of thyroid gland | rs78269692 | 0.06 | 0.07 | 1.058 (0.930-1.205) | 3.92E-01 |
| Overall Breast Cancer | Benign neoplasm of thyroid gland | rs78440108 | 0.05 | 0.07 | 1.056 (0.927-1.204) | 4.12E-01 |
| Overall Breast Cancer | Benign neoplasm of thyroid gland | rs78540526 | 0.03 | 0.07 | 1.028 (0.900-1.174) | 6.85E-01 |
| Overall Breast Cancer | Benign neoplasm of thyroid gland | rs7939702 | 0.05 | 0.07 | 1.052 (0.923-1.199) | 4.49E-01 |
| Overall Breast Cancer | Benign neoplasm of thyroid gland | rs7971 | 0.05 | 0.07 | 1.051 (0.922-1.198) | 4.53E-01 |
| Overall Breast Cancer | Benign neoplasm of thyroid gland | rs79724016 | 0.05 | 0.07 | 1.051 (0.922-1.198) | 4.55E-01 |
| Overall Breast Cancer | Benign neoplasm of thyroid gland | rs8105994 | 0.06 | 0.07 | 1.065 (0.935-1.213) | 3.44E-01 |
| Overall Breast Cancer | Benign neoplasm of thyroid gland | rs889310 | 0.05 | 0.07 | 1.053 (0.924-1.200) | 4.41E-01 |
| Overall Breast Cancer | Benign neoplasm of thyroid gland | rs941764 | 0.05 | 0.07 | 1.048 (0.920-1.194) | 4.83E-01 |
| Overall Breast Cancer | Benign neoplasm of thyroid gland | rs9611271 | 0.05 | 0.07 | 1.048 (0.920-1.193) | 4.84E-01 |
| Overall Breast Cancer | Benign neoplasm of thyroid gland | rs9620778 | 0.05 | 0.07 | 1.051 (0.922-1.198) | 4.60E-01 |
| Overall Breast Cancer | Benign neoplasm of thyroid gland | rs970822 | 0.05 | 0.07 | 1.049 (0.920-1.195) | 4.76E-01 |
| Overall Breast Cancer | Benign neoplasm of thyroid gland | rs9712235 | 0.05 | 0.07 | 1.053 (0.924-1.200) | 4.40E-01 |
| Overall Breast Cancer | Benign neoplasm of thyroid gland | rs9833888 | 0.05 | 0.07 | 1.055 (0.925-1.202) | 4.25E-01 |
| Overall Breast Cancer | Benign neoplasm of thyroid gland | rs9952980 | 0.05 | 0.07 | 1.049 (0.921-1.196) | 4.69E-01 |
| Overall Breast Cancer | Benign neoplasm of thyroid gland | rs9954058 | 0.05 | 0.07 | 1.052 (0.923-1.199) | 4.46E-01 |
| Overall Breast Cancer | Benign neoplasm of thyroid gland | All | 0.05 | 0.07 | 1.052 (0.924-1.199) | 4.44E-01 |

**Table S8. The result of leave-one-out approach for Malignant neoplasm of thyroid gland and Overall Breast Cancer**

| **Exposure** | **Outcome** | **SNP** | **Beta** | **Standard error** | **OR (95%CI)** | **P-value** |
| --- | --- | --- | --- | --- | --- | --- |
| Malignant neoplasm of thyroid gland | Overall Breast Cancer | rs10817378 | 0.09 | 0.06 | 1.097 (0.969-1.242) | 1.42E-01 |
| Malignant neoplasm of thyroid gland | Overall Breast Cancer | rs10982766 | 0.10 | 0.06 | 1.108 (0.979-1.255) | 1.05E-01 |
| Malignant neoplasm of thyroid gland | Overall Breast Cancer | rs17293443 | 0.10 | 0.07 | 1.101 (0.966-1.254) | 1.50E-01 |
| Malignant neoplasm of thyroid gland | Overall Breast Cancer | rs2373058 | 0.07 | 0.06 | 1.073 (0.949-1.214) | 2.60E-01 |
| Malignant neoplasm of thyroid gland | Overall Breast Cancer | rs2755193 | 0.09 | 0.07 | 1.099 (0.967-1.249) | 1.47E-01 |
| Malignant neoplasm of thyroid gland | Overall Breast Cancer | rs4129579 | 0.10 | 0.06 | 1.105 (0.974-1.254) | 1.20E-01 |
| Malignant neoplasm of thyroid gland | Overall Breast Cancer | rs62237573 | 0.05 | 0.04 | 1.056 (0.983-1.134) | 1.39E-01 |
| Malignant neoplasm of thyroid gland | Overall Breast Cancer | rs7034310 | 0.11 | 0.06 | 1.112 (0.981-1.261) | 9.73E-02 |
| Malignant neoplasm of thyroid gland | Overall Breast Cancer | rs722082 | 0.07 | 0.06 | 1.071 (0.946-1.213) | 2.80E-01 |
| Malignant neoplasm of thyroid gland | Overall Breast Cancer | All | 0.09 | 0.06 | 1.091 (0.974-1.222) | 1.33E-01 |

**Table S9. The result of leave-one-out approach for Benign neoplasm of thyroid gland and Overall Breast Cancer**

| **Exposure** | **Outcome** | **SNP** | **Beta** | **Standard error** | **OR (95%CI)** | **P-value** |
| --- | --- | --- | --- | --- | --- | --- |
| Benign neoplasm of thyroid gland | Overall Breast Cancer | rs10983655 | -0.01 | 0.02 | 0.986 (0.954-1.019) | 4.12E-01 |
| Benign neoplasm of thyroid gland | Overall Breast Cancer | rs12266386 | -0.01 | 0.02 | 0.987 (0.957-1.018) | 4.15E-01 |
| Benign neoplasm of thyroid gland | Overall Breast Cancer | rs13259143 | -0.02 | 0.02 | 0.981 (0.953-1.011) | 2.10E-01 |
| Benign neoplasm of thyroid gland | Overall Breast Cancer | rs4815130 | -0.01 | 0.02 | 0.985 (0.956-1.016) | 3.45E-01 |
| Benign neoplasm of thyroid gland | Overall Breast Cancer | All | -0.02 | 0.01 | 0.985 (0.959-1.012) | 2.66E-01 |
